# Development and validation of a postpartum cardiovascular disease risk prediction model in women incorporating reproductive and pregnancy-related predictors

**DOI:** 10.1186/s12916-025-04229-1

**Published:** 2025-08-29

**Authors:** Steven Wambua, Francesca L. Crowe, Shakila Thangaratinam, Dermot O’Reilly, Colin McCowan, Sinead Brophy, Christopher Yau, Krishnarajah Nirantharakumar, Richard D. Riley, Kym I. E. Snell, the MuM-PreDiCT Group

**Affiliations:** 1 Department of Applied Health Sciences, College of Medicine and Health, University of Birmingham, Edgbaston, Birmingham, UK; 2 National Institute for Health and Care Research (NIHR) Birmingham Biomedical Research Centre, Birmingham, UK; 3 Institute of Metabolism and Systems Research, WHO Collaborating Centre for Global Women’s Health, University of Birmingham, Birmingham, UK; 4 Department of Obstetrics and Gynaecology, Birmingham Women’s and Children’s NHS Foundation Trust, Birmingham, UK; 5 Centre for Public Health, Queen’s University Belfast, Belfast, UK; 6 School of Medicine, University of St Andrews, St Andrews, UK; 7 Data Science, Medical School, Swansea University, Swansea, UK; 8 Big Data Institute, Li Ka Shing Centre for Health Information and Discovery, University of Oxford, Old Road Campus, Oxford, OX3 7LF UK; 9 Nuffield Department of Women’s & Reproductive Health, Level 3 Women’s Centre, John Radcliffe Hospital, University of Oxford, Oxford, OX3 9DU UK; 10 Health Data Research, London, UK

**Keywords:** Risk prediction, Pregnancy complications, QRISK®-3, Cardiovascular disease

## Abstract

**Background:**

Each year, over 700,000 pregnancies occur in the UK, with up to 10% affected by complications such as hypertensive disorders of pregnancy and gestational diabetes mellitus. Pregnancy-related complications and reproductive factors are associated with an increased risk of cardiovascular disease (CVD) later in life. Our aim was to determine whether adding pregnancy factors to a prediction model with established CVD risk factors improves 10-year risk prediction of CVD in postpartum women, using QRISK®-3 as a benchmark model.

**Methods:**

We used a population-based retrospective cohort of women aged 15 to 49 who had been pregnant from the Clinical Practice Research Datalink (CPRD) primary care database. Women who were CVD-free were followed from 6 months postpartum. We evaluated the performance of QRISK®-3 and updated the risk prediction model using established risk factors for CVD from QRISK®-3 and additional risk factors specific to pregnancy. Models were developed using Cox-proportional hazards regression for CVD within 10 years. Models were evaluated and compared using measures of overall model fit, calibration, discrimination and clinical utility.

**Results:**

Among 567,667 eligible women, 2175 (0.38%) experienced a CVD event within 10 years. The median follow-up was 4 years. Of the additional pregnancy factors, gestational hypertension, preeclampsia, miscarriage, stillbirth, postnatal depression, gravidity, endometriosis and polycystic ovary syndrome remained associated with CVD after adjusting for other established risk factors of CVD. Adding pregnancy factors to those from QRISK®-3 led to marginal improvements in model performance (QRISK®-3 *C*-statistic: 0.703 (95% CI 0.687 to 0.718), *updated model C-statistic*: 0.726 (95% CI 0.711 to 0.740) Although calibration did not improve overall, subgroup analysis showed better calibration in women with a history of pre-eclampsia, postnatal depression and preterm birth using the updated model. The clinical utility was improved for updated models.

**Conclusions:**

The updated risk prediction models resulted in marginal improvement in discrimination and calibration compared to QRISK®-3 in postpartum women. This could be due to the known association of pregnancy-related complications with established risk factors of CVD. Although the overall predictive performance and calibration of the updated models was similar, the updated model resulted in better clinical utility.

**Supplementary Information:**

The online version contains supplementary material available at 10.1186/s12916-025-04229-1.

## Background

Cardiovascular disease is a leading cause of morbidity and mortality globally [1, 2]. Identifying individuals at a higher risk of developing CVD can help clinicians in decision making and provide information to patients for accessing preventive treatments to reduce their risk. There have been efforts to actualize this through the development of risk prediction models such as the Framingham risk score and the QRISK® score [3, 4]. Studies have, however, reported that these algorithms developed for the general population might underestimate the risk of CVD in young women [5, 6]. Although the algorithms include several traditional risk factors for CVD (e.g. diabetes, family history of CVD), they do not include factors related to pregnancy complications for women despite existing evidence showing that pregnancy complications (e.g. hypertensive disorders of pregnancy, placental abruption, preterm birth, gestational diabetes mellitus, stillbirth) and reproductive factors e.g. early age at menarche and polycystic ovary syndrome) are associated with the risk of CVD in women [7].

Although the postpartum period has been identified as a window of opportunity to initiate preventive measures to reduce the risk of CVD in this population [8], the value of pregnancy and reproductive-related factors in the risk prediction modelling for CVD in the postpartum period has received little attention until recently. Results from studies have shown only a slight improvement in the predictive performance of risk prediction models developed using predictors from two CVD risk prediction models (NORRISK 2 risk model (improved the c-index by 0.004) and the Framingham risk score (improved the c-index by 0.0053)) after including pregnancy complications in the models [9, 10]. Although recent evidence shows several reproductive and pregnancy-related factors are associated with increased risk of CVD, there has been no attempt to assess the value of pregnancy and reproductive-related factors added to those of QRISK®3, the algorithm used to assess a person’s 10-year risk of CVD in the general UK population.

Although recent evidence shows a marginal improvement in NORRISK 2 and the Framingham risk scores when pregnancy complications are included, these studies had important limitations that constrain their applicability. For instance, e.g. in NORRISK study did not include GDM, a recognised predictor of CVD, and highlighted this omission as a limitation. The generalisability of its findings is also restricted, as the cohort was limited to women 40 years and older, and while smoking rates in the study were similar to the average among Norwegian women, overall smoking prevalence in Norway is higher than other higher-income countries, limiting international relevance. Similarly, although the study evaluating the Framingham score included several pregnancy complications, it did not consider reproductive factors such as PCOS, endometriosis and menstrual irregularity all of which are associated with increased risk of CVD. The study was conducted among a general population that resided in district 13 of Tehran, and while reported as representative of an urban Iranian population, it may not be generalisable to broader or more diverse populations. In addition, the study relied on complete case analysis, excluding individuals with missing data, which may introduce bias and limit robustness.

In summary, although both NORRISK2 and Framingham scores were developed to predict the risk of CVD similar to QRISK3, they differ in terms of the population characteristics used to develop the models and the range of candidate predictors considered. Our study builds on this evidence by evaluating the added value of pregnancy and reproductive factors, some of which have not been considered in previous studies, within QRISK®−3, the risk equation developed and implemented in the UK population. This work provides new insights into the potential for improving CVD risk prediction using routinely collected healthcare data from a nationally representative cohort.

To our knowledge, only one recent study has attempted to incorporate new predictors related to women to QRISK®−3 [11]. The study considered miscarriage, placental abruption, pre-eclampsia, postnatal depression, gestational diabetes, endometriosis and PCOS. However, the study did not consider other factors such as gestational hypertension, preterm birth, stillbirth, small for gestational age, gravidity and menstrual irregularity which are associated with increased risk of CVD [12]. In addition, the study included only pre-eclampsia and postpartum depression in the final model, excluding other pregnancy-related factors because of a lack of statistically significant associations. The study was also conducted in a general population of women, rather than specifically among those who have been pregnant. As a result, the findings may not be generalisable to the target population in which evaluating of pregnancy-related factors is likely to be more relevant.

The main objective of this study was to assess whether adding reproductive health and pregnancy-related candidate predictors to those of the QRISK®−3 risk score improves the performance of the individual risk prediction of CVD in women who had been pregnant. The specific objectives were to:

i) Externally validate the QRISK®−3 equation in our target population
ii) Update the model and internally validate it
  - Using the QRISK®−3 linear predictor as a single predictor and consider additional candidate predictors (Model 1).
  - Using all predictors from QRISK®−3 plus additional candidate predictors (Model 2a).

## Methods

### Data source

The Clinical Practice Research Datalink (CPRD) Gold database, which has over 19 million patient records in the UK from over 940 participating general practices, was used. The CPRD pregnancy register, which captures information from maternity, antenatal, and delivery records, was used to identify pregnancies within CPRD GOLD.

### Study population

The target population was women who had been pregnant aged 15 to 49 years who were registered with their GPs between January 2000 and December 2021 with linkage to the Hospital Episodes Statistics (HES). To ensure sufficient quality data at baseline, participants contributed to the cohort after a minimum registration period with their practice of at least a year. Women were followed up from 15 months after date of conception (approximately 6 months postpartum) of the current pregnancy (i.e. for women with more than one pregnancy, the last pregnancy was used), regarded as the index date, to allow for normal physiological changes of pregnancy to resolve and allow time for postpartum information to be recorded in the primary care database [13, 14]. Women were followed until the earliest of outcome date (diagnosis of cardiovascular disease), transfer date from the practice, last date of practice data collection, date of death or study end date. In the absence of any of the above events, participants were censored 10 years after the index date. Women with pre-existing CVD or on statins before the index date were excluded.

### Predictor variables

#### Traditional predictors

The traditional risk factors of CVD were obtained from the QRISK®3 algorithm [15]. These were age, ethnicity, deprivation (quintiles of Townsend score), systolic blood pressure (SBP), standard deviation of at least two SBP measurements, body mass index (BMI), total/HDL cholesterol ratio, smoking status, family history of CVD in a first degree relative aged less than 60, diabetes, rheumatoid arthritis, atrial fibrillation, chronic kidney disease, diagnosis of migraine, corticosteroid use, systemic lupus erythematosus, atypical antipsychotics, current treatment for hypertension (at least one of thiazide, β blocker, calcium channel blocker, or angiotensin converting enzyme inhibitor), and diagnosis of severe mental illness. Similar to QRISK®−3, medications (treatment for hypertension, corticosteroids and atypical antipsychotics) were measured as at least two prescriptions before the index date with the latest prescription recorded within 28 days of the index date. For all the other predictors, the latest information recorded in the general practice before the index date was obtained.

#### Additional pregnancy-related candidate predictors

Several pregnancy and reproductive-related factors were identified from an umbrella review on the associations of reproductive factors with CVD and from discussions with clinicians and patient research partners [12]. These included polycystic ovary syndrome, pre-eclampsia, small for gestational age, postnatal depression, endometriosis, irregular menses, gestational diabetes mellitus, gestational hypertension, miscarriage, preterm birth, placental abruption and number of previous pregnancies [16]. The pregnancy-related candidate predictors were measured as any history of the pregnancy complication from previous pregnancies (e.g. history of gestational diabetes mellitus before the current/last pregnancy).

All candidate predictors were evaluated to quantify missing data, identify outliers and ensure the correct measurement units were used. Definitions of the candidate predictors are provided in Additional file 1: Table 1 [17–19].

### Outcome

The outcome of this study was the first recorded diagnosis of cardiovascular disease (coronary heart disease, stroke, myocardial infarction, or transient ischemic attack). This definition was based on the QRISK®−3 algorithm’s definition of CVD to ensure comparability of the updated models [20].

### Statistical analysis

#### Missing data

For the external validation of QRISK®−3, the approach used to handle missing data at the implementation of the algorithm was adopted. Missing systolic blood pressure, body mass index, and total/HDL cholesterol ratio were imputed based on age and sex using single imputation in line with recommendations from the recent literature [21, 22]. Missing smoking status was assumed to be non-smoker, ethnicity was assumed to be white, and missing deprivation scores were imputed using the median value. Missing entry of a condition was taken to indicate absence of the condition (e.g. missing diabetes record was taken to mean no diabetes).

For the development of updated models, candidate predictors with more than 40% missing data were excluded; otherwise, the above single imputation approach was used. A table with proportion missing for each variable and method of handling the missing data is provided in Additional file 1: Table 2.

#### Evaluation of QRISK®−3 in external data

The first objective was to evaluate the QRISK®−3 algorithm in the population of women who had been pregnant to assess the performance of the risk equation in this cohort. This formed the benchmark for models with additional pregnancy-related predictors.

We calculated the 10-year predicted risk of CVD in the cohort using the QRISK®−3 women’s risk Eq. [15]. The 10-year observed risk was obtained using a pseudo-value approach [23]. The performance of the model was then evaluated using measures of discrimination (the model’s ability to differentiate between those who developed CVD and those who did not) and calibration (agreement between predicted and observed risk). Discrimination was quantified using Harrell’s *C* statistic, time-dependent *C* statistic and Royston’s *D* statistic. Calibration was quantified by plotting the 10-year observed probability of CVD against the 10-year predicted probability of CVD using the “pmcalplot” package in Stata using the default 10 equal risk groups based on percentiles [24]. In addition, summary measures of calibration (calibration-in-the-large, calibration slope and calibration intercept) were estimated. Mean calibration (calibration-in-the-large), which measures the agreement between predicted and observed survival probability, was estimated as the ratio of the observed survival probability (Kaplan–Meier estimate of experiencing CVD at 10 years) and the average predicted risk at 10 years [25]. The calibration intercept was calculated by fitting a generalized linear model of pseudo-values as the outcome and the predicted risk estimates (transformed with complementary log–log function) as an offset. The intercept from this model indicates the predicted risk is too high if the intercept is negative and too low if the intercept is positive [26, 27]. The calibration slope was estimated by fitting a similar model to that used for the calibration intercept but allowing the coefficient for the (complementary log–log) transformed predicted risks to be estimated. The coefficient of the transformed predicted risk estimates is the calibration slope [26, 27].

The clinical utility of the model was assessed using decision curves considering a range of risk thresholds up to 10% [28, 29]. We used the ‘dcurves’ package to visualize net benefit and plotting the decision curve. We used vector of threshold probabilities between 0 and 1 with the default sequence by 0.01 [28, 30].

#### Model update: re-calibrating the baseline risk of QRISK®−3

To assess whether the predictive performance of QRISK®−3 could be improved by re-estimating the baseline risk in the cohort of younger postpartum women, we re-calibrated QRISK®−3 using the 10-year baseline survival value estimated in the cohort by forcing the predictor effects to be the same (fitting the survival data to the QRISK®−3 linear predictor as an offset using Cox regression model) and re-assessed the performance of the re-calibrated model.

#### Model development and evaluation of updated models

After evaluating the performance of the QRISK®−3 algorithm (the benchmark model), three new models were developed and internally validated; Model 1 included the QRISK®−3 linear predictor (obtained from external validation step) plus pregnancy-related factors as predictors, Model 2a included QRISK®−3 predictors only (without interaction terms) to re-estimate QRISK®−3 coefficients and lastly Model 2b included QRISK®−3 predictors plus pregnancy related factors.

The primary timepoint of interest for the risk prediction models was 10 years in line with NICE guideline recommendations for interventions based on the 10-year risk of CVD [31].

Cox proportional hazards regression was used to develop the new models following practical approaches for risk prediction models [32, 33]. The accompanying 10-year baseline survival for each model was estimated non-parametrically using the Breslow method. The initial model included all the candidate predictors, and then variable selection was performed using the least absolute shrinkage and selection operator (LASSO) to determine predictors included in each model [34, 35]. The QRISK®−3 predictors were forced to remain in the model. After variables were selected, the final model was then fitted using Cox regression with the selected additional predictors. The continuous variables were included in the models on their continuous scale, with non-linear relationships with the outcome modelled using fractional polynomial terms. The fractional polynomial terms for the continuous variables were obtained based on complete data similar to QRISK development [15] and the resulting terms were then used in developing the updated models, including variable selection using LASSO. Internal validation was performed using 500 bootstrap samples to account for overfitting and estimate optimism, repeating the modelling process in each bootstrap sample and comparing performance in the bootstrap sample and original data to obtain optimism-adjusted statistics. Measures of discrimination and calibration were used to evaluate the new models and were compared with the performance of QRISK®−3. All analyses were conducted in R statistical software, R version 4.2.1 and in Stata.

### Sample size

Determination of sample size for external validation and development of the new models was detailed in the protocol for this study [16]. Briefly, we established that a minimum sample size of about 24,000 women and 264 CVD events would result in precise estimates of model performance, for example with a calibration slope CI width of 0.3 (i.e. CI width of 0.85–1.15 assuming the true value is 1), with an assumed 20% censoring rate by 10 years [36, 37].

### Sensitivity analysis

Because QRISK®−3 was developed for those aged 25 to 84 years, we carried out sensitivity analysis to compare the performance of QRISK®−3 with and without women aged below 25 years to assess the impact of applying the model outside the age group included in the development of the model. We also repeated the analysis in complete data (patients without missing data in the predictors). We also repeated the analysis after using multiple imputation with chained equations to impute variables with missing data. Multivariable imputation with chained equations was performed to generate 20 imputed datasets for missing BMI, SBP, total cholesterol: HDL cholesterol ratio (TC: HDL), systolic blood pressure (SBP), SBP standard deviation and smoking status. Performance measures were pooled across the imputed datasets using Rubin’s rules [38].

### Model presentation

This study has been reported following the Transparent Reporting of a Multivariable Prediction Model for Individual Prognosis or Diagnosis (TRIPOD + AI) guidelines (Additional file 1: Table 3) [20, 39].

## Results

### Cohort description

**Fig. 1:**
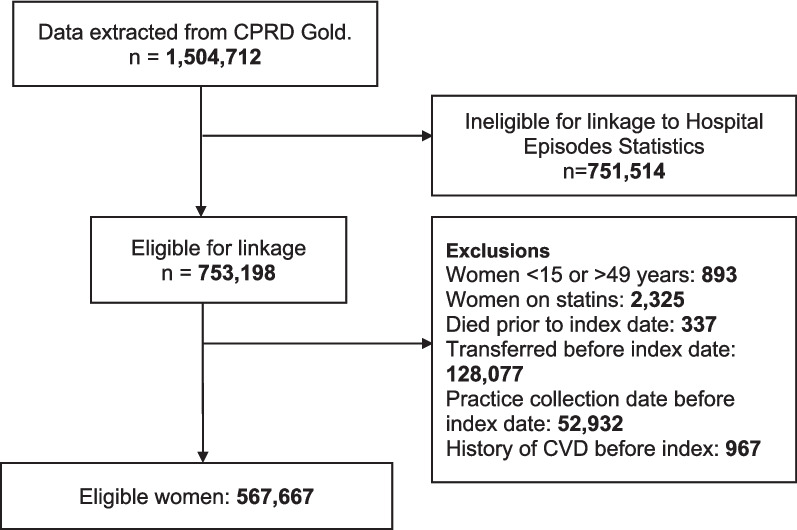
Flow diagram of number of patients in the study cohort

Overall, 1,504,712 women with the current pregnancy between 2000 and 2021 were identified in CPRD Gold pregnancy register. Of these, only 753,198 women were eligible for linkage to the Hospital Episodes Statistics (HES) based on eligibility flag within HES database. The following were excluded at start of follow up; 893 were outside the age range of interest (15 to 49 years), 2325 were prescribed statins, 337 died prior to the index date, 128,077 had left their practice before index date, 52,932 had last data collection from their practice before index date and 967 had a history of CVD. Overall, 567,667 women were included in developing the risk prediction model out of which 5558 (0.98%) were diagnosed with CVD during follow-up. Figure 1 presents the flow diagram of the inclusion criteria.

Table 1 presents the baseline characteristics of the derivation cohort. The mean age of women was 32 years and the mean BMI was 25.68 kg/m^2^ at the start of follow-up. Most women in the cohort were white ethnicity (81%) and non-smokers (66.1%). Migraine (9.0%) was the most common comorbidity in the cohort. The following predictors had missing data; SBP (16%), SBP variability (40%), BMI (24%), total cholesterol/HDL ratio (91%), smoking status (18%) and Townsend (0.11%). SBP variability and total cholesterol/HDL ratio were used for the external validation of QRISK®−3 and excluded from the derivation of Model 2a and Model 2b because of the high level of missing data.

**Table 1:** Baseline characteristics of women who had been pregnant aged 15 to 49 years included in the study at cohort entry from CPRD GOLD who were registered with their GPs between January 2000 and December 2021

	**Summary statistics**
**Variable**	
Total	567,667
Mean (SD) age, years	32.01 (6.63)
Mean (SD) systolic blood pressure	115.16 (12.72)
Missing (%) systolic blood pressure	87,970 (16)
Median (IQR) systolic blood pressure standard deviation	8.37 [5.51, 10.98]
Missing (%) systolic blood pressure standard deviation	224,504 (40)
Median (IQR) total cholesterol/HDL ratio	3.23 [2.67, 3.96]
Missing (%) total cholesterol/HDL ratio	516,752 (91)
Family history of CVD *n* (%)	18,351 (3.2)
**Ethnicity categories for QRISK®−3 validation** ***n*** **(%)**	
White or unknown	477,906 (84.2)
Indian	14,892 (2.6)
Pakistani	8984 (1.6)
Bangladeshi	2856 (0.5)
Other Asian	10,299 (1.8)
Black Caribbean	5637 (1.0)
Black African	17,715 (3.1)
Chinese	3596 (0.6)
Other ethnicity	25,782 (4.5)
**CPRD Ethnicity categories** ***n*** **(%)**	
White or unknown	453,253 (79.8)
Black	42,547 (7.5)
Asian	35,390 (6.2)
Mixed	8026 (1.4)
Others	28,451 (5.0)
**Deprivation score**	
Median (IQR) Townsend	3.00 [2.00, 4.00]
Missing (%) Townsend	611 (0.11)
Mean (SD) body mass index, kg/m^2^	25.68 (5.71)
Missing (%) body mass index	134,497 (24)
**Smoking status levels** ***n*** **(%)**	
Non-smoker or unknown	374,510 (66.0)
Ex-smoker	85,820 (15.1)
Light smoker	107,313 (18.9)
Moderate smoker	14 (< 0.1)
Heavy smoker	10 (< 0.1)
**Health conditions** ***n*** **(%)**	
Atrial fibrillation	368 (0.1)
Migraine	51,278 (9.0)
Rheumatoid arthritis	1335 (0.2)
Chronic kidney disease (stage 3, 4 or 5)	391 (0.1)
Severe mental illness	5338 (0.9)
Systemic lupus erythematosus	677 (0.1)
Type 1 diabetes	1489 (0.3)
Type 2 diabetes	2357 (0.4)
**Medications** ***n*** **(%)**	
Atypical antipsychotics	1563 (0.3)
Corticosteroids	1530 (0.3)
Treated hypertension	1785 (0.3)
**Pregnancy complications** ***n*** **(%)**	
Hypertensive disorders of pregnancy (pre-eclampsia)	18,676 (3.3)
Hypertensive disorders of pregnancy (gestational hypertension)	16,956 (3.0)
Small for gestational age	34,935 (6.2)
Postnatal depression	30,732 (5.4)
Gestational diabetes mellitus	16,257 (2.9)
Stillbirth	5254 (0.9)
Miscarriage	127,020 (22.4)
Preterm birth	23,525 (4.1)
Placental abruption	2954 (0.5)
**Reproductive factors** ***n*** **(%)**	
Endometriosis	12,515 (2.2)
Irregular menses	67,587 (11.9)
Polycystic ovary syndrome	21,306 (3.8)
Median (IQR) Gravidity	2.00 [1.00, 3.00]
**Gravidity** ***n*** **(%)**	
1	256,305 (45.2)
2	154,547 (27.2)
3	81,358 (14.3)
4	40,785 (7.2)
≥ 5	34,672 (6.1)
**Follow-up time and CVD events**	
Median (IQR) follow-up time, years	3.66 [1.39, 7.78]
CVD events (%)	1613 (0.28)

*Abbreviations*: *CVD* cardiovascular disease, *HDP* hypertensive disorders of pregnancy, *HDL* high-density lipoprotein cholesterol, *IQR* interquartile range, *SD* standard deviation

Pregnancy complications with more than 1% prevalence were miscarriage (20.2%), small for gestational age (6.2%), preterm birth (4.1%), gestational hypertension (3.0%), pre-eclampsia (3.3%) and Gestational diabetes mellitus (GDM) (2.9%) and reproductive factors of interest were irregular menses (11.9%), postnatal depression (5.4%), polycystic ovary syndrome (PCOS) (3.8%) and endometriosis (2.2%).

### Model development

Table 2 shows the adjusted hazard ratios (HR) in the development cohort. Only four pregnancy-related factors were dropped during variable selection in Model 2b (preterm birth, small for gestational age, irregular menses and placental abruption) and no candidate predictor was dropped in the other models. In both Model 1 and Model 2b, where pregnancy-related factors were included, most of the pregnancy and reproductive factors were associated with an increased risk of CVD. In Model 1, pre-eclampsia, gestational diabetes, postnatal depression, gestational hypertension, miscarriage, preterm birth, stillbirth, and gravidity were positively associated with the risk of CVD (pre-eclampsia HR 1.53 (95% CI 1.25 to 1.87), GDM HR 1.21 (95% CI 0.97 to 1.50), postnatal depression HR 1.36 (95% CI 1.14 to 1.62), Gestational hypertension HR 1.36 (95% CI 1.09 to 1.71), miscarriage HR 1.11 (95% CI 1.00 to 1.25), pre-term birth HR 1.18 (95% CI 0.96 to 1.45), stillbirth HR 1.41 (95% CI 1.01 to 1.96), placental abruption HR 1.25 (95% CI 0.73 to 2.12)), gravidity (HR 1.07 (95% 1.05 to 1.10), endometriosis HR 1.49 (95% CI 1.17 to 1.90), irregular menses HR 1.05 (95% CI 0.91 to 1.21) and polycystic ovary syndrome (PCOS) HR 1.42 (95% CI 1.14 to 1.76). In Model 2b, a similar direction of effect was observed. The results from the complete case analysis (Additional file 1: Table 4), restricting the data to those aged 25 years and above (Additional file 1: Table 5) and when using multiple imputation for missing variables (Additional file 1: Table 6 were similar.

**Table 2:** Adjusted hazard ratios (95% confidence interval) for cardiovascular disease in the models with and without additional pregnancy and reproductive-related predictors

Predictors	Model 1 HR (95% CI)	Model 2a HR (95% CI)	Model 2b HR (95% CI)
**QRISK3 linear predictor (LP)**			
LP 1^§^	1.06 (1.05 to 1.07)	-	-
LP 2^§^	0.98 (0.97 to 0.98)	-	-
**Established risk factors**			
Age^§^	-	2.17 (2.00 to 2.36)	2.04 (1.87 to 2.22)
BMI^§^	-	1.36 (1.25 to 1.48)	1.29 (1.18 to 1.41)
SBP^§^	-	1.94 (1.68 to 2.24)	1.83 (1.58 to 2.11)
Townsend scores	-	1.18 (1.14 to 1.23)	1.18 (1.14 to 1.23)
Family history of CVD	-	1.52 (1.22 to 1.89)	1.48 (1.19 to 1.84)
**Ethnicity**			
Ref: White ethnicity	-	1	1
Black ethnicity	-	1.55 (1.34 to 1.79)	1.54 (1.34 to 1.78)
Mixed ethnicity	-	1.21 (0.78 to 1.89)	1.24 (0.80 to 1.94)
Asian ethnicity	-	1.21 (0.97 to 1.50)	1.19 (0.96 to 1.48)
Other ethnicity	-	0.89 (0.70 to 1.13)	0.90 (0.70 to 1.14)
**Smoking status**			
Ref: non smoker	-	1	1
Ex smoker	-	0.91 (0.78 to 1.07)	0.89 (0.76 to 1.04)
Current smoker	-	1.78 (1.58 to 2.00)	1.73 (1.54 to 1.94)
**Health conditions**			
Atrial fibrillation	-	2.79 (1.14 to 6.86)	2.79 (1.13 to 6.92)
Migraine	-	1.70 (1.48 to 1.95)	1.58 (1.38 to 1.82)
Rheumatoid arthritis	-	1.07 (0.52 to 2.20)	1.02 (0.50 to 2.11)
Chronic kidney disease (stage 3, 4 or 5)	-	4.37 (2.15 to 8.84)	3.82 (1.88 to 7.77)
Severe mental illness	-	2.01 (1.43 to 2.82)	1.89 (1.34 to 2.64)
SLE	-	3.98 (2.26 to 6.99)	3.50 (1.98 to 6.17)
Diabetes Type 1	-	5.84 (3.92 to 8.70)	5.30 (3.54 to 7.94)
Diabetes Type 2	-	4.51 (3.44 to 5.92)	3.94 (2.96 to 5.25)
**Medications**			
Atypical antipsychotics	-	1.00 (0.49 to 2.06)	0.98 (0.48 to 2.01)
Corticosteroids	-	1.89 (1.14 to 3.14)	1.67 (1.00 to 2.77)
Antihypertensives	-	1.52 (1.05 to 2.19)	1.32 (0.91 to 1.91)
**Pregnancy factors**			
Pre-eclampsia	1.53 (1.25 to 1.87)	-	1.52 (1.24 to 1.86)
Postnatal depression	1.36 (1.14 to 1.62)	-	1.33 (1.11 to 1.58)
Gestational diabetes mellitus	1.21 (0.97 to 1.50)	-	1.18 (0.94 to 1.48)
Gestational hypertension	1.36 (1.09 to 1.71)	-	1.34 (1.07 to 1.69)
Miscarriage	1.11 (1.00 to 1.25)	-	1.16 (1.03 to 1.29)
Preterm birth	1.18 (0.96 to 1.45)	-	-
Stillbirth	1.41 (1.01 to 1.96)	-	1.37 (0.99 to 1.91)
Small for gestational age	0.90 (0.71 to 1.14)	-	-
Gravidity	1.07 (1.04 to 1.10)	-	1.07 (1.05 to 1.10)
**Reproductive factors**			
Endometriosis	1.49 (1.17 to 1.90)	-	1.56 (1.22 to 1.99)
Polycystic ovary syndrome	1.42 (1.14 to 1.76)	-	1.41 (1.13 to 1.75)
Irregular menses	1.05 (0.91 to 1.21)	-	-
Placental abruption	1.25 (0.73 to 2.12)	-	-

*Abbreviations*: *CVD* cardiovascular disease, *SLE* systemic lupus erythematosus, *IQR* interquartile range, *SD* standard deviation

^§^Fractional polynomial of the predictor: age: (age/10), body mass index (BMI 1): (BMI/10), systolic blood pressure (SBP 1): (SBP/100)^2, QRISK®−3 linear predictor 1^§^: (linear predictor + 4)^3, QRISK®−3 linear predictor 2^§^: (linear predictor + 4)^3 × logarithm((linear predictor + 4))

### Discrimination, calibration and clinical utility

Table 3 shows the predictive performance measures of the models in the development cohort. Adding pregnancy factors and reproductive factors to those of QRISK®−3 resulted in marginal improvement in discrimination of 10-year risk of CVD (QRISK®−3 C statistic 0.703 (95% CI 0.687 to 0.718), Model 1 C statistic 0.715 (95% CI 0.700 to 0.730), Model 2a C statistic 0.717 (95% CI 0.702 to 0.732), Model 2b C statistic 0.726 (95% CI 0.711 to 0.740). The optimism-adjusted C statistics for Model 1, Model 2a and Model 2b were 0.711, 0.713 and 0.720. The D statistic was also higher in models with additional pregnancy factors QRISK®−3 D statistic 1.357 (95% CI (1.275 to 1.439), Model 1 D statistic 1.462 (95% CI 1.379 to 1.545), Model 2a D statistic 1.409 (95% CI 1.329 to 1.489), Model 2b D statistic 1.498 (95% CI 1.417 to 1.579). Performance of the models by age groups, ethnicity categories and reproductive and pregnancy complications are presented in Additional file 1: Table 7a–7c.

**Table 3:** Model performance measures in models with QRISK®−3 predictors only and models with QRISK®−3 predictors plus pregnancy and reproductive related factors

Model	Harrell’s *C*	Royston’s *D*	*R*^2^*D*	Distribution of LP, mean (SD)
QRISK3	0.703 (0.687 to 0.718)	1.357 (1.275 to 1.439)	0.3055	−1.123 (0.955)
Model 1	0.715 (0.700 to 0.730)	1.462 (1.379 to 1.545)	0.3378	0.110 (0.741)
Model 2a	0.717 (0.702 to 0.732)	1.409 (1.329 to 1.489)	0.3215	0.222 (0.732)
Model 2b	0.726 (0.711 to 0.740)	1.498 (1.417 to 1.579)	0.3487	0.310 (0.780)

There was no substantial difference in the performance measures in the sensitivity analyses based on complete case analysis (Additional file 1: Tables 8 and 9a–9c), restricting the analysis to those aged 25 years of age and above (Additional file 1: Tables 10 and 11a–11c) and using multiple imputation to handle missing data (Additional file 1: Table 12).

Table 4 shows the results of calibration-in-the-large (mean calibration), calibration intercept and slope for the prediction models. The observed/expected ratio (O/E) was close to one for all the updated models, and the calibration intercept for all the models was also close to zero, indicating good calibration as expected. The calibration slopes for all the models were closer to 1 (ideal agreement between observed and predicted risks).

**Table 4:** Mean, intercept and slope of calibration

Model	O/E (95% CI)	Intercept	Slope
QRISK3	1.346 (1.282 to 1.413)	0.192 (0.102 to 0.281)	0.872 (0.820 to 0.924)
Model 1	1.085 (1.033 to 1.139)	− 0.018 (− 0.112 to 0.076)	1.00 (0.950 to 1.050)
Model 2a	1.059 (1.008 to 1.112)	− 0.022 (− 0.113 to 0.070)	1.00 (0.951 to 1.049)
Model 2b	1.051 (1.001 to 1.104)	− 0.047 (− 0.142 to 0.049)	1.00 (0.953 to 1.047)

**Fig. 2:**
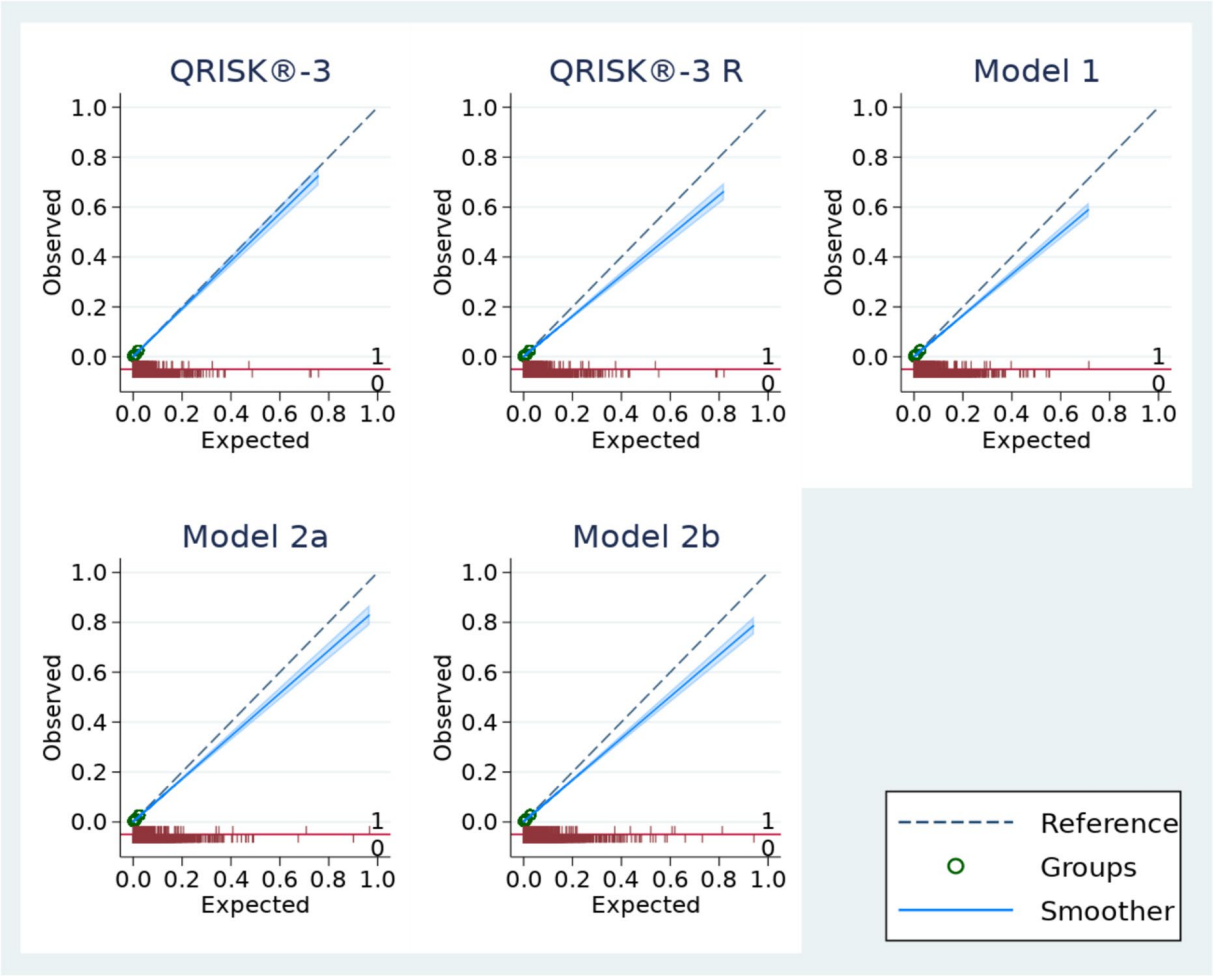
Calibration curves

Figure 2 displays the calibration curves of agreement between the 10-year observed and predicted CVD risk for all models. The plot suggests the QRISK®−3 equation was very well calibrated in predicting 10-year CVD risk for the cohort of women who had been pregnant across all the risk thresholds. The recalibrated QRISK®−3 equation, Model 1, Model 2a, and Model 2b were well calibrated for lower risk thresholds and overestimated CVD risk for higher observed risks. The QRISK®−3 equation and the recalibrated model were better calibrated for older age groups, and the updated models were better calibrated in younger age groups (Additional file 1: Fig. S1a–S1b). The QRISK®−3 equation was well calibrated in women from white and black ethnicities and was miscalibrated in women of Asian, mixed and other ethnicities. The calibration plots from some of the ethnicity groups should be interpreted with caution as the number of events in the women from Asian, mixed and other ethnicities was few (1176 from white ethnicity, 239 from black ethnicity, 92 from Asian ethnicity, 20 from mixed ethnicity, 71 from other ethnicity and 15 from unknown ethnicity). The updated models resulted in similar calibration as QRISK®−3 in women from white ethnicity, better calibration than QRISK3 in women of Asian ethnicity, and miscalibration in women from black, mixed and other ethnicities (Additional file 1: Fig. S2a–S2f). In comparison to QRISK3, the updated models had better calibration in women with a history of preterm birth and postpartum depression. We also observed QRISK3 underestimated risk in women with a history of pre-eclampsia, preterm birth and postnatal depression (Additional file 1: Fig. S3a–S3l).

Sensitivity analyses restricting analysis to complete data (Additional file 1: Fig. S4, S5a–S5b, S6a–S6f, S7a–Sl) and to those aged 25 years and above (Additional file 1: Fig. S8, S9a–S9b and S10a–S10f) and when using multiple imputation for missing variables (Additional file 1: Fig. S14) showed similar patterns in calibration.

**Fig. 3:**
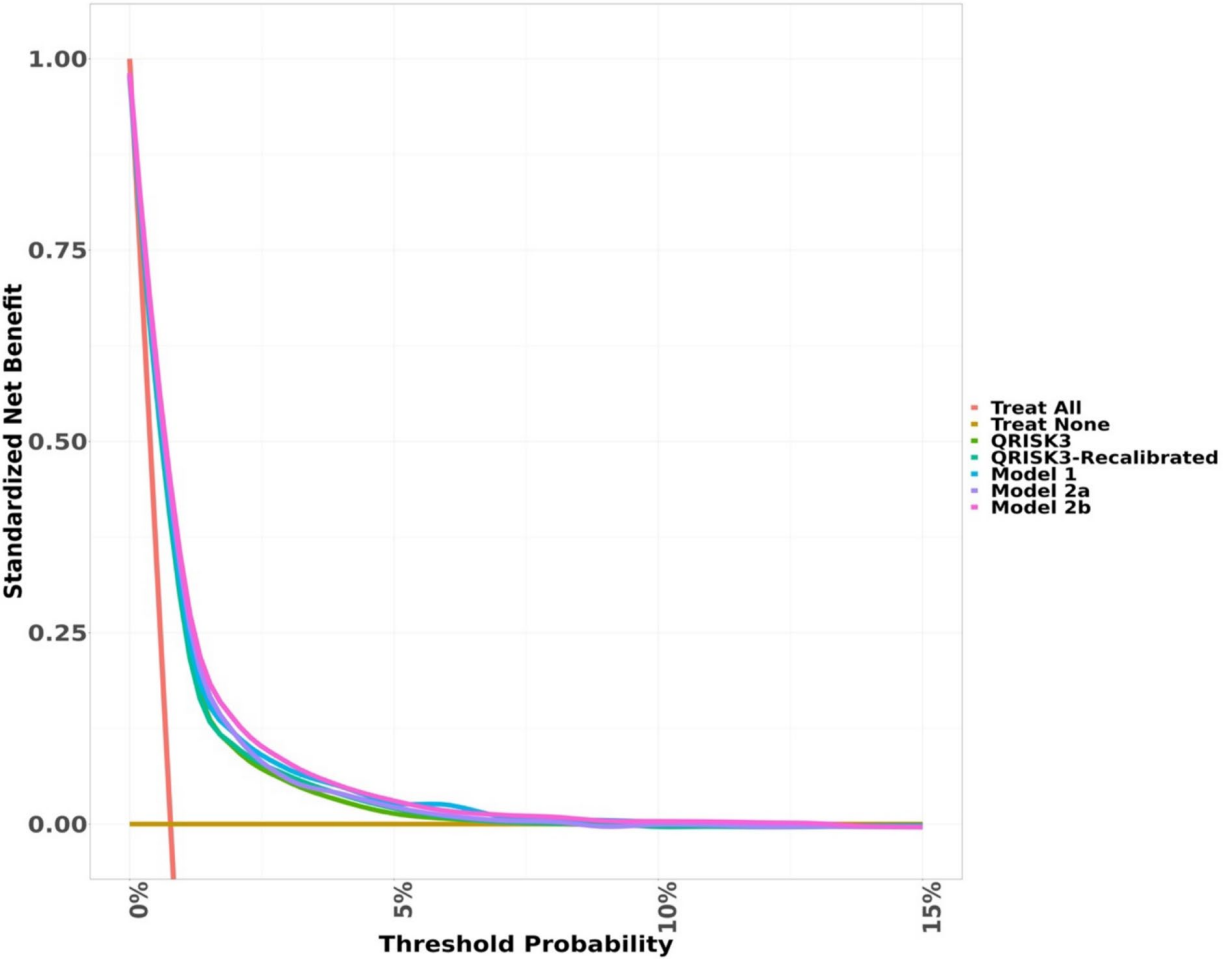
Decision curve

Figure 3 shows the decision curve of the net benefit of implementing QRISK®−3 equation and the updated models in the cohort of women who had been pregnant. The net benefit of all the models is higher than “treat all” and “treat none” interventions. The updated models showed slightly higher net benefit compared to QRISK®−3, and model 2b was marginally better than all the models. Sensitivity analyses restricting analysis to complete data (Additional file 1: Fig. S13), restricting to those aged 25 years and above (Additional file 1: Fig. S14) and using multiple imputation for missing variables (Additional file 1: Fig. S15) showed similar clinical utility of the models.

## Discussion

### Principal findings

In this study, we have independently evaluated the QRISK®−3 equation for 10-year risk of CVD in women aged 15 to 49 years who have been pregnant. We have also updated QRISK®−3 for this population by developing and internally validating three further models: Model 1, which included the QRISK®−3 linear predictor and the addition of pregnancy factors; Model 2a, which re-estimated the coefficients for the QRISK®−3 predictors using our cohort; and Model 2b, which included QRISK®−3 predictors and pregnancy-related factors.

The discrimination of QRISK®−3 was moderate and the calibration plots indicated good calibration overall in the cohort of women who had been pregnant. Model 1 resulted in moderate discrimination and good calibration in lower risk thresholds with overestimation of risk for higher observed risks. Both Model 2a and 2b resulted in moderate discrimination and excellent calibration for lower risks and overestimation of risk for higher observed risks, showing that adding pregnancy factors to established risk factors of CVD did not result in substantially better discrimination and calibration. However, analysis of the clinical utility of the models in the cohort of postpartum women showed the models with additional pregnancy factors resulted in higher net benefit across risk thresholds compared to the models without the pregnancy factors.

### Comparison with literature

Although this is the first study to evaluate the value of pregnancy complications and reproductive factors in 10-year CVD prediction using QRISK®−3 as a benchmark, previous studies have explored the same question using other established CVD prediction tools as benchmarks [9, 10, 40, 41]. In a study that evaluated the added value of including pregnancy complications history in 10-year CVD risk prediction in women aged 50 or 60 years in Sweden, history of pregnancy complications (hypertensive disorders of pregnancy and low birth weight) was associated with increased risk of CVD but did not meaningfully improve 10-year CVD risk prediction when compared with a reference lab-based model published [41]. Similarly, a recent study that sought to assess the value of adding history of placenta previa, placenta abruption, preterm birth, miscarriage, stillbirth, HDP, GDM and ectopic pregnancy to established predictors of CVD in the Framingham CVD risk equation in women aged 30 to 70 years led to a small improvement in discrimination (0.0053 increase in the *C*-statistic) [10].

In another study, inclusion of pre-eclampsia, gestational hypertension, preterm birth and small for gestational age in established risk factors of CVD from the NORRISK2 prediction model (the reference model) did not improve CVD prediction in women aged 40 years and older from two primary care hospitals in Norway [9, 42]. The study found that after adjusting for established risk factors of CVD, only pre-eclampsia was associated with increased risk of CVD but adding pregnancy complications to NORRISK 2 predictors led to a small improvement in the discrimination (0.004 increase in the c-index) and no difference in the calibration of the models.

Similar findings have also been reported in a study that evaluated the additional value of including HDP and parity to traditional risk factors of CVD in predicting 10-year risk of CVD in women aged 40 years and older from a questionnaire-based prospective study of nurses in the US using an established CVD risk equation as a reference [40].

Similar to a recent study that considered miscarriage, placental abruption, pre-eclampsia, postnatal depression, gestational diabetes, endometriosis and PCOS in the general population of women, we found pre-eclampsia and postpartum depression to be associated with CVD after accounting for other established risk factors of CVD [11]. Although the study did not find association between miscarriage, endometriosis and PCOS, we found these factors to be associated with increased risk of CVD after accounting for established risk factors of CVD. These differences could be attributed to the differences in the population for which these factors were evaluated as our study was conducted specifically among those who have been pregnant, as a result, the findings from the previous study may not be generalisable to the target population in which evaluating of pregnancy-related factors is likely to be more relevant.

Our study is also in line with a previous systematic review on pregnancy complication history in the 10-year CVD risk prediction, which found that established CVD risk prediction models are not meaningfully improved by incorporating pregnancy-related factors as predictors in the models [43].

Other studies evaluated CVD risk prediction for shorter time points. A study that evaluated the added value of HDP and GDM on the 1-year risk of CVD using three established CVD risk scores as benchmarks reported small improvements in the risk prediction [44].

Moderate performance of CVD risk prediction models in women of reproductive age has also been reported in previous studies [45, 46].

### Interpretation

The findings that including information of pregnancy history to the existing prediction models marginally improves their predictive performance. Previous research has also reported that the C statistic is insensitive to addition of new candidate predictors in a model, even if the new predictors are statistically significant or clinically significant and hence the C statistic is generally not a useful measure in evaluating new risk factors [47]. However, a better measure in this context would be calibration. The marginal improvement in discrimination could be because of known association of pregnancy complications with established risk factors of CVD. For example, women with pre-eclampsia have higher blood pressure and are more likely to develop chronic hypertension compared to women without pre-eclampsia. Since treatment for hypertension is an established predictor of CVD, including history of HDP does not add more information to the CVD risk prediction [48]. Previous studies have also found the association between HDP and risk of CVD is mediated by established risk factors of CVD such as chronic hypertension, type 2 diabetes and overweight/obesity [49]. Moreover, studies have consistently reported that being overweight or obese increases the risk of type 2 diabetes and hypertension with obesity-related risk factors such as insulin resistance and inflammation contributing to the pathogenesis of these conditions [50, 51].

### Strengths and weaknesses

This study offers the first external validation of QRISK®−3 in women who had been pregnant in the UK. The study has employed robust analytical techniques for assessing performance of QRISK®−3 by considering a systematic way of assessing the added value of additional pregnancy factors on the discrimination, calibration, and clinical utility of the QRISK®−3 in the low-risk population of young women. We have also carried out several sensitivity analyses to assess the impact of missing data and including women younger than 25 years in QRISK®−3 implementation. This study also follows best practices in development and validation of clinical risk prediction models and contributes more knowledge regarding 10-year CVD risk prediction in women who had been pregnant. The study benefits from a large cohort of women across many general practices in the UK.

Although we provide valuable insights in this study, we acknowledge the following limitations. Firstly, although the CPRD Gold database is representative of the UK population, there is variation in primary care clinical coding rates across general practices. Secondly, although we used various methods for handling missing data, various mechanisms of missingness could be at play such as data missing not at random, and hence informative missingness. For example, if a biomarker test such as blood cholesterol has been carried out, then the perceived need for the test might be informative of the patient’s health [22]. Thirdly, the median follow-up in the study cohort overall might not be enough to effectively evaluate the risk of CVD at 10 years, and the few CVD events in subgroups of interest such as ethnicity and age groups lead to performance measures with large uncertainty and should be interpreted with caution. We also note some candidate predictors identified from literature associated with CVD in women of reproductive age, such as early menarche, early menopause and infertility history, were not considered in this study because they are not well captured in primary care records and may be more appropriate for consideration in women of reproductive age who have not been pregnant. Lastly, predictors of CVD related to genetics, which could potentially improve the prediction models, were not included in this study because there is still little information in primary care datasets.

### Future research

More research could focus on externally validating our model in women with a history of pregnancy complications as it performed better in women with complications such as pre-eclampsia, postnatal depression and pre-term birth and further evaluate the influence of specific pregnancy factors on the predictive performance of the models and potentially propose specific updates to current models for women with these specific pregnancy complications to enhance local performance. Further research could evaluate the impact of variability in various general practices on the performance of the risk prediction models using random-effects models as event rates and management of conditions varies by practice and this could have an impact on the baseline risk of patients in different practices. Future research could also consider external validation in datasets with longer follow-up and including time-dependent analysis. Further, developing risk tools for cardiometabolic renal conditions (including type 2 diabetes, hypertension and chronic kidney disease in addition to CVD) which include pregnancy complications using multistate modelling frameworks to further understand other pregnancy complications associated with the risk of these conditions as they have similar management and clinical pathways. The clinical utility of models with pregnancy complications was better compared to models without the factors and studies to evaluate and demonstrate the feasibility of introducing CVD risk assessment for women with a history of pregnancy complications into clinical practice to detect CVD in the postpartum period could be conducted.

## Conclusions

Although updated risk prediction models resulted in better discrimination and calibration compared to QRISK®−3 in the cohort of women who had been pregnant, adding pregnancy and reproductive history to established risk factors of CVD did not substantially improve discrimination and calibration of the risk prediction models in the low-risk population of young women. This could be due to the known association of pregnancy-related complications with established risk factors of CVD, and similar findings have been reported in other studies. Although the overall predictive performance and calibration of the updated models are similar, the model with additional factors results in better clinical utility in women with a history of pregnancy, and more research could be done to evaluate the feasibility of incorporating CVD screening in these women after pregnancy.

## Supplementary Information

Additional file 1: Tables 1–12 and Fig. S1-S15. Table 1 – Definitions of variables, Table 2 – Missing data proportions in each variable, Table 3- Transparent Reporting of a multivariable prediction model for Individual Prognosis Or Diagnosis + Artificial Intelligence TRIPOD + AI checklist, Table 4—Hazard ratios from sensitivity analysis based on complete case analysis, Table 5—Hazard ratios from sensitivity analysis restricting the data to those aged 25 years of age and above, Table 6: Table 6: Hazard ratios from sensitivity analysis using multiple imputation for missing data, Table 7a – Discrimination statics by age groups from primary analysis, Table 7b – Discrimination statics by Ethnicity from primary analysis, Table 7c – Discrimination statics by reproductive factors and pregnancy complications for primary analysis, Table 8– Overall discrimination in sensitivity analysis based on complete case analysis, Table 9a- Discrimination statics by age groups for complete case analysis, Table 9b – Discrimination statics by Ethnicity for complete case analysis, Table 9c – Discrimination statics by reproductive factors and pregnancy complications for complete case analysis Table 10—Overall discrimination in sensitivity analysis restricting to those aged 25 years of age and above, Table 11a- Discrimination statics by age groups when we restricted the analysis to those aged 25 years and above, Table 11b- Discrimination statics by Ethnicity when we restricted the analysis to those aged 25 years and above, Table 11c- Discrimination statics by reproductive factors and pregnancy complications when we restricted the analysis to those aged 25 years and above, Table 12—Overall discrimination in sensitivity analysis using multiple imputation, Fig. S1a- Calibration plots for primary analysis in those aged 15 to 34 years, Fig. S1b- Calibration plots for primary analysis in those aged 35 to 49 years, Fig. S2a- Calibration plots for primary analysis in white or unknown ethnicity, Fig. S2b- Calibration plots for primary analysis in Black ethnicity, Fig. S2c- Calibration plots for primary analysis in Asian ethnicity, Fig. S2d- Calibration plots for primary analysis in Mixed ethnicity, Fig. S2e- Calibration plots for primary analysis in Other ethnicity, Fig. S2f- Calibration plots for primary analysis in unknown ethnicity, Fig. S3a- Calibration plots for primary analysis in women with a history of PCOS, Fig. S3b- Calibration plots for primary analysis in women with a history of irregular menses, Fig. S3c- Calibration plots for primary analysis in women with a history of Endometriosis, Fig. S3d- Calibration plots for primary analysis in women with a history of miscarriage, Fig. S3e- Calibration plots for primary analysis in women with a history of gestational hypertension, Fig. S3f- Calibration plots for primary analysis in women with a history of pre-eclampsia, Fig. S3g- Calibration plots for primary analysis in women with a history of GDM, Fig. S3h- Calibration plots for primary analysis in women with a history of preterm birth, Fig. S3i- Calibration plots for primary analysis in women with a history of Stillbirth, Fig. S3j- Calibration plots for primary analysis in women with a history of placental abruption, Fig. S3k- Calibration plots for primary analysis in women with a history of SGA, Fig. S3l- Calibration plots for primary analysis in women with a history of postnatal depression Fig. S4- Overall calibration plots for complete case analysis, Fig. S5a- Calibration plots for complete case analysis in those aged 15 to 34 years, Fig. S5b- Calibration plots for complete case analysis in those aged 35 to 49 years, Fig. S6a- Calibration plots for primary analysis in white or unknown ethnicity, Fig. S6b- Calibration plots for complete case analysis in Black ethnicity, Fig. S6c- Calibration plots for complete case analysis in Asian ethnicity, Fig. S6d- Calibration plots for complete case analysis in Mixed ethnicity, Fig. S6e- Calibration plots complete case analysis in Other ethnicity, Fig. S7a- Calibration plots for complete case analysis in women with a history of PCOS, Fig. S7b- Calibration plots for complete case analysis in women with a history of irregular menses, Fig. S7c- Calibration plots for complete case analysis in women with a history of Endometriosis, Fig. S7d- Calibration plots for complete case analysis in women with a history of miscarriage, Fig. S7e- Calibration plots for complete case analysis in women with a history of gestational hypertension, Fig. S7f- Calibration plots for complete case analysis in women with a history of pre-eclampsia, Fig. S7g- Calibration plots for complete case analysis in women with a history of GDM, FigS7h- Calibration plots for complete case analysis in women with a history of preterm birth, Fig. S7i- Calibration plots for complete case analysis in women with a history of Stillbirth, Fig. S7j- Calibration plots for complete case analysis in women with a history of placental abruption, Fig. S7k- Calibration plots for complete case analysis in women with a history of SGA, Fig. S7l- Calibration plots for complete case analysis in women with a history of postnatal depression, Fig. S8- Overall calibration plots for sensitivity analysis in those aged 25 and above, Fig. S9a- Calibration plots for sensitivity analysis in those aged 25 and above in those aged 25 to 34 years, Fig. S9b- Calibration plots for sensitivity analysis in those aged 25 and above in those aged 35 to 49 years, Fig. S10a- Calibration plots for sensitivity analysis in those aged 25 and above in white or unknown ethnicity, Fig. S10b- Calibration plots for sensitivity analysis in those aged 25 and above in Black ethnicity, Fig. S10c- Calibration plots for sensitivity analysis in those aged 25 and above in Asian ethnicity, Fig. S10d- Calibration plots for sensitivity analysis in those aged 25 and above in Mixed ethnicity, Fig. S10e- Calibration plots sensitivity analysis in those aged 25 and above in Other ethnicity. Fig. S10f- Calibration plots sensitivity analysis in those aged 25 and above in unknown ethnicity. Fig. S11a- Calibration plots for those aged 25 and above in women with a history of PCOS, Fig. S11b- Calibration plots for those aged 25 and above in women with a history of irregular menses, Fig. S11c- Calibration plots for those aged 25 and above in women with a history of Endometriosis, Fig. S11d- Calibration plots for those aged 25 and above in women with a history of miscarriage, Fig. S11e- Calibration plots for those aged 25 and above in women with a history of gestational hypertension, Fig. S11f- Calibration plots for those aged 25 and above in women with a history of pre-eclampsia, Fig. S11g- Calibration plots for those aged 25 and above in women with a history of GDM, Fig. S11h- Calibration plots for those aged 25 and above in women with a history of preterm birth, Fig. S11i- Calibration plots for those aged 25 and above women with a history of Stillbirth, Fig. S11j- Calibration plots for those aged 25 and above in women with a history of placental abruption, Fig. S11k- Calibration plots for those aged 25 and above in women with a history of SGA, Fig. S11l- Calibration plots for those aged 25 and above in women with a history of postnatal depression Fig. S12- Overall calibration plots for sensitivity analysis using multiple imputation for missing data, Fig. S13- Clinical utility in complete case analysis, Fig. S14- Clinical utility in those aged 25 years and above, Fig. S15- Clinical utility in analysis based on multiple imputation.

## Data Availability

The datasets used for this study are available from CPRD and can be accessed upon reasonable request. Supplementary resources are also provided.

## Abbreviations

BMI: Body mass index
CI: Confidence intervals
CPRD: Clinical Practice Research Datalink
CVD: Cardiovascular disease
GDM: Gestational diabetes mellitus
GP: General practitioner
HDL: High-density lipoprotein
HDP: Hypertensive disorders of pregnancy
HES: Hospital Episodes Statistics
HR: Hazard ratio
IQR: Interquartile range
LASSO: Least absolute shrinkage and selection operator
LP: Linear predictor
MuM-PreDiCT: Multimorbidity in Pregnancy: Determinants, clusters, Consequences and Trajectories
NHS: National Health Service
NICE: The National Institute for Health and Care Excellence
PCOS: Polycystic ovary syndrome
SBP: Systolic blood pressure
SD: Standard deviation
SLE: Systemic lupus erythematosus
T2DM: Type 2 diabetes mellitus
TRIPOD + AI: Transparent Reporting of a multivariable prediction model for Individual Prognosis Or Diagnosis + Artificial Intelligence
