# Supplementary material for: Development and validation of a postpartum cardiovascular disease risk prediction model in women incorporating reproductive and pregnancy-related predictors
Source: BMC Med. 2025 Aug 29;23:508. doi: 10.1186/s12916-025-04229-1 (PMC12398157; doi:10.1186/s12916-025-04229-1)
Supplement: Supplementary file 1 — Additional file 1: Tables 1–12 and Fig. S1-S15. Table 1 – Definitions of variables, Table 2 – Missing data proportions in each variable, Table 3- Transparent Reporting of a multivariable prediction model for Individual Prognosis Or Diagnosis + Artificial Intelligence TRIPOD + AI checklist, Table 4—Hazard ratios from sensitivity analysis based on complete case analysis, Table 5—Hazard ratios from sensitivity analysis restricting the data to those aged 25 years of age and above, Table 6: Table 6: Hazard ratios from sensitivity analysis using multiple imputation for missing data, Table 7a – Discrimination statics by age groups from primary analysis, Table 7b – Discrimination statics by Ethnicity from primary analysis, Table 7c – Discrimination statics by reproductive factors and pregnancy complications for primary analysis, Table 8– Overall discrimination in sensitivity analysis based on complete case analysis, Table 9a- Discrimination statics by age groups for complete case analysis, Table 9b – Discrimination statics by Ethnicity for complete case analysis, Table 9c – Discrimination statics by reproductive factors and pregnancy complications for complete case analysis Table 10—Overall discrimination in sensitivity analysis restricting to those aged 25 years of age and above, Table 11a- Discrimination statics by age groups when we restricted the analysis to those aged 25 years and above, Table 11b- Discrimination statics by Ethnicity when we restricted the analysis to those aged 25 years and above, Table 11c- Discrimination statics by reproductive factors and pregnancy complications when we restricted the analysis to those aged 25 years and above, Table 12—Overall discrimination in sensitivity analysis using multiple imputation, Fig. S1a- Calibration plots for primary analysis in those aged 15 to 34 years, Fig. S1b- Calibration plots for primary analysis in those aged 35 to 49 years, Fig. S2a- Calibration plots for primary analysis in white or unknown ethnicity, Fig. S [file 12916_2025_4229_MOESM1_ESM.docx]

Table of Contents

[Additional file 1	2](#_Toc200286819)

[Table 1: Variables definition	3](#_Toc200286820)

[Table 2: Missing data	5](#_Toc200286821)

[Table 3: TRIPOD+AI checklist	6](#_Toc200286822)

[Table 4: Hazard ratios from sensitivity analysis based on complete case analysis (CCA)	8](#_Toc200286823)

[Table 5: Hazard ratios from sensitivity analysis restricting the data to those aged 25 years of age and above	9](#_Toc200286824)

[Table 6: Hazard ratios from sensitivity analysis using multiple imputation for missing data	10](#_Toc200286825)

[Table 7: Discrimination by sub-groups in main analysis	12](#_Toc200286826)

[Table 7a: Age groups	12](#_Toc200286827)

[Table 7b: Ethnicity	12](#_Toc200286828)

[Table 7c: Reproductive and pregnancy complications	13](#_Toc200286829)

[Table 8: Overall discrimination in sensitivity analysis based on complete case analysis	15](#_Toc200286830)

[Table 9a: Discrimination by Age groups in complete case analysis	16](#_Toc200286831)

[Table 9b: Discrimination by Ethnicity in complete case analysis	17](#_Toc200286832)

[Table 9c: Discrimination by Reproductive and pregnancy complications in complete case analysis	18](#_Toc200286833)

[Table 10: Overall discrimination in sensitivity analysis restricting to those aged 25 years of age and above	21](#_Toc200286834)

[Table 11a: Discrimination by Age groups in sensitivity analysis restricting to those aged 25 years of age and above	21](#_Toc200286835)

[Table 11b: Discrimination by Ethnicity in sensitivity analysis restricting to those aged 25 years of age and above	22](#_Toc200286836)

[Table 11c: Discrimination by reproductive factors and pregnancy complications	23](#_Toc200286837)

[Table 12: Overall discrimination in sensitivity analysis using multiple imputation	25](#_Toc200286838)

[Calibration plots by subgroup in main analysis	26](#_Toc200286839)

[Fig. S1: Calibration plots by Age groups in main analysis	26](#_Toc200286840)

[Fig. S2: Calibration plots by Ethnicity in main analysis	28](#_Toc200286841)

[Fig. S3: Calibration plots by Reproductive and pregnancy complications in main analysis	34](#_Toc200286842)

[Calibration plots overall and by subgroup in complete case analysis	46](#_Toc200286843)

[Fig. S4: Overall Calibration plot	46](#_Toc200286844)

[Fig. S5: Age groups	47](#_Toc200286845)

[Fig. S6: Ethnicity	49](#_Toc200286846)

[Fig. S7: Reproductive and pregnancy complications	54](#_Toc200286847)

[Calibration plots overall and by subgroup in those aged 25 years and above	66](#_Toc200286848)

[Fig. S8: Overall calibration plot	66](#_Toc200286849)

[Fig. S9: Age groups	67](#_Toc200286850)

[Fig. S10: Ethnicity	69](#_Toc200286851)

[Fig. S11: Reproductive and pregnancy complications	75](#_Toc200286852)

[Calibration in sensitivity analysis based on multiple imputation	87](#_Toc200286853)

[Fig. S12: Calibration plot in sensitivity analysis based on multiple imputation	87](#_Toc200286854)

[Clinical utility in complete case analysis	88](#_Toc200286855)

[Fig. S13: Clinical utility in complete case analysis	88](#_Toc200286856)

[Clinical utility in those aged 25 years and above	89](#_Toc200286857)

[Fig. S14: Clinical utility	89](#_Toc200286858)

[Clinical utility in sensitivity analysis based on multiple imputation	90](#_Toc200286859)

[Fig. S15: Clinical utility	90](#_Toc200286860)

[Appendix	91](#_Toc200286861)

[Sensitivity analysis: Counting number of pregnancy complications	91](#_Toc200286862)

[Table 13: Hazard Ratios	91](#_Toc200286863)

[Table 14: Discrimination	92](#_Toc200286864)

[Fig. S16: Calibration	92](#_Toc200286865)

[Fig. S17: Clinical utility	93](#_Toc200286866)

[References	94](#_Toc200286867)

# Additional file 1

## Table 1: Variables definition

| Variable |  |  |
| --- | --- | --- |
| Continuous variable | Age | Age at baseline |
|  | Body Mass Index | Latest record before index date |
|  | Systolic Blood Pressure | Latest record before index date |
|  | Systolic blood pressure standard deviation | Standard deviation of SBP if woman has more than one SBP record before index date |
|  | Total cholesterol:HDL cholesterol (TC:HDL) ratio | Latest record before index date |
| Categorical variables | Ethnicity | Last recorded code before index date. To obtain the nine categories of ethnicity in QRISK3, we used medical codes from a previous study that externally validated QRISK3 (15). We used these together with ethnicity categories provided by CPRD and linked to Hospital Episodes Statistics. The CPRD ethnicity categories we used –Asian, Black, Mixed, Others, White and Unknown – are based on the classifications from the Census of England and Wales (16) where the mixed ethnicity category is defined as (White and Black Caribbean, White and Black African, White and Asian, or Any other Mixed or multiple ethnic background). |
|  | Smoking status | Latest recorded before index date. Smoking status was defined using medical codes as (non-smoker, ex-smoker, light smoker (1-9 cigarettes/day), moderate smoker (10-19 cigarettes/day), or heavy smoker (≥20 cigarettes/day)), similar to QRISK3. |
|  | Townsend score | Quintiles of Townsend score provided through linked data to HES |
| Medications | Antihypertensive medication (Treated hypertension) | At least two prescriptions of antihypertensive agent, with the most recent one no more than 28 days before index date similar to QRISK3. |
|  | Corticosteroids | At least two prescriptions of Corticosteroids, with the most recent one no more than 28 days before index date similar to QRISK3. |
|  | Atypical antipsychotics | At least two prescriptions of atypical antipsychotic medication, with the most recent one no more than 28 days before index date similar to QRISK3. |
| Family history | Family history of coronary artery disease in first degree relative <60 years | Present if ever recorded before index date |
| Conditions | Type 1 diabetes | Present if ever recorded before patient study entry date using medical codes. |
|  | Type 2 diabetes | Present if ever recorded before patient study entry date using medical codes. |
|  | Rheumatoid arthritis | Present if ever recorded before patient study entry date using medical codes. |
|  | Atrial fibrillation | Present if ever recorded before patient study entry date using medical codes. |
|  | Chronic kidney disease (stage 3, 4 or 5) | Present if ever recorded before patient study entry date using medical codes. |
|  | Migraine | Present if ever recorded before patient study entry date using medical codes. |
|  | Systemic lupus erythromatosus | Present if ever recorded before patient study entry date using medical codes. |
|  | Severe mental illness (including psychosis, schizophrenia, or bipolar affective disease) | Present if ever recorded before patient study entry date using medical codes. |
| Reproductive factors | Endometriosis | Present if ever recorded before patient study entry date using medical codes. |
|  | Irregular menses (menstrual cycle irregularity) | Present if ever recorded before patient study entry date using medical codes. Diagnostic Read code describing menstrual cycle irregularity or frequent or infrequent cycles as reported in primary care were used similar to a previous study within our team (17). |
|  | Polycystic ovary syndrome | Present if ever recorded before patient study entry date using medical codes. |
|  | Gravidity | Number of pregnancies recorded in the pregnancy register |
| Pregnancy complications | Pre-eclampsia | Present if ever recorded before patient study entry date using medical codes. |
|  | Gestational hypertension | Present if ever recorded before patient study entry date using medical codes. |
|  | Small for gestational age | Present if ever recorded before patient study entry date using medical codes. We also used the INTERGROWTH-21^st^ software to calculate weight centiles and SGA was defined as weight centile below than 10. |
|  | Postnatal depression | Present if ever recorded before patient study entry date using medical codes. |
|  | Gestational diabetes mellitus | Present if ever recorded before patient study entry date using medical codes. |
|  | Stillbirth | Present if ever recorded before patient study entry date using medical codes. We also used the stillbirth outcome flag from pregnancy register. |
|  | Miscarriage | Present if ever recorded before patient study entry date using medical codes. We also used the miscarriage outcome flag from pregnancy register. |
|  | Preterm birth | Present if ever recorded before patient study entry date using medical codes. We also used the preterm birth outcome flag from pregnancy register. |
|  | Placental abruption | Present if ever recorded before patient study entry date using medical codes. |

**Github links to medical codes used within the MuM-Predict consortium where this study was carried out.**

<https://github.com/mumpredict/Exposure-multimorbidity-clinical-codes->

<https://github.com/mumpredict/Read-codes-and-ICD-10-codes>

<https://github.com/mumpredict/Outcome-clinical-code-lists>

## Table 2: Missing data

| **Variable** | **How missingness  was handled  in analysis** | **Eligible cohort   N=567,667  No (%) missing data** |
| --- | --- | --- |
| Age | Never missing | 0 |
| Body mass index (BMI) | Imputed | 134,497 (24) |
| Total cholesterol:HDL cholesterol (TC:HDL) ratio | Imputed | 516,752 (91) |
| Systolic blood pressure | Imputed | 87,970 (16) |
| Systolic blood pressure standard deviation | Imputed | 224,504 (40) |
| Smoking status | Imputed with median | 101087 (18) |
| Ethnicity | Assumed to be white | 18652 (3) |
| Townsend | Imputed with median | 611 (0.1) |
| Condition/reproductive/pregnancy complications variables | Assumed to be absent if no record | NA |

## Table 3: TRIPOD+AI checklist

| **Section/Topic Item Development Checklist item**  **/ evaluation**^1^ | | | | **Reported on page** |
| --- | --- | --- | --- | --- |
| **TITLE** | | | |  |
| *Title* | 1 | D;E | Identify the study as developing or evaluating the performance of a multivariable prediction model, the target population, and the outcome to be predicted | 1 |
| **ABSTRACT** | | | | |
| *Abstract* | 2 | D;E | See TRIPOD+AI for Abstracts checklist | 2 |
| **INTRODUCTION** | | | | |
| *Background* | 3a | D;E | Explain the healthcare context (including whether diagnostic or prognostic) and rationale for developing or evaluating the prediction model, including references to existing models | 3-4 |
|  | 3b | D;E | Describe the target population and the intended purpose of the prediction model in the context of the care pathway, including its intended users (e.g., healthcare professionals, patients, public) | 3-4 |
|  | 3c | D;E | Describe any known health inequalities between sociodemographic groups | 3-4 |
| *Objectives* | 4 | D;E | Specify the study objectives, including whether the study describes the development or validation of a prediction model (or both) | 4 |
| **METHODS** | | | | |
| *Data* | 5a | D;E | Describe the sources of data separately for the development and evaluation datasets (e.g., randomised trial, cohort, routine care or registry data), the rationale for using these data, and representativeness of the data | 4 |
|  | 5b | D;E | Specify the dates of the collected participant data, including start and end of participant accrual; and, if applicable, end of follow-up | 4 |
| *Participants* | 6a | D;E | Specify key elements of the study setting (e.g., primary care, secondary care, general population)  including the number and location of centres | 4 |
|  | 6b | D;E | Describe the eligibility criteria for study participants | 4-5 |
|  | 6c | D;E | Give details of any treatments received, and how they were handled during model development or evaluation, if relevant | NA |
| *Data preparation* | 7 | D;E | Describe any data pre-processing and quality checking, including whether this was similar across  relevant sociodemographic groups | 5-6 |
| *Outcome* | 8a | D;E | Clearly define the outcome that is being predicted and the time horizon, including how and when assessed, the rationale for choosing this outcome, and whether the method of outcome assessment is  consistent across sociodemographic groups | 6 |
|  | 8b | D;E | If outcome assessment requires subjective interpretation, describe the qualifications and demographic characteristics of the outcome assessors | NA |
|  | 8c | D;E | Report any actions to blind assessment of the outcome to be predicted | NA |
| *Predictors* | 9a | D | Describe the choice of initial predictors (e.g., literature, previous models, all available predictors) and  any pre-selection of predictors before model building | 5-6 |
|  | 9b | D;E | Clearly define all predictors, including how and when they were measured (and any actions to blind assessment of predictors for the outcome and other predictors) | 5-6 |
|  | 9c | D;E | If predictor measurement requires subjective interpretation, describe the qualifications and demographic characteristics of the predictor assessors | NA |
| *Sample size* | 10 | D;E | Explain how the study size was arrived at (separately for development and evaluation), and justify that  the study size was sufficient to answer the research question. Include details of any sample size calculation | 9 |
| *Missing data* | 11 | D;E | Describe how missing data were handled. Provide reasons for omitting any data | 6 |
| *Analytical methods* | 12a | D | Describe how the data were used (e.g., for development and evaluation of model performance) in the analysis, including whether the data were partitioned, considering any sample size requirements | 7-9 |
|  | 12b | D | Depending on the type of model, describe how predictors were handled in the analyses (functional form,  rescaling, transformation, or any standardisation). | 7-9 |
|  | 12c | D | Specify the type of model, rationale^2^, all model-building steps, including any hyperparameter tuning,  and method for internal validation | 8-9 |
|  | 12d | D;E | Describe if and how any heterogeneity in estimates of model parameter values and model performance was handled and quantified across clusters (e.g., hospitals, countries). See TRIPOD-Cluster for  additional considerations^3^ | NA |
|  | 12e | D;E | Specify all measures and plots used (and their rationale) to evaluate model performance (e.g., discrimination, calibration, clinical utility) and, if relevant, to compare multiple models | 7-9 |
|  | 12f | E | Describe any model updating (e.g., recalibration) arising from the model evaluation, either overall or for particular sociodemographic groups or settings | 8-9 |
|  | 12g | E | For model evaluation, describe how the model predictions were calculated (e.g., formula, code, object, application programming interface) | 9 |
| *Class imbalance* | 13 | D;E | If class imbalance methods were used, state why and how this was done, and any subsequent methods to  recalibrate the model or the model predictions | NA |
| *Fairness* | 14 | D;E | Describe any approaches that were used to address model fairness and their rationale | NA |
| *Model output* | 15 | D | Specify the output of the prediction model (e.g., probabilities, classification). Provide details and  rationale for any classification and how the thresholds were identified | 14 |

^1^ D=items relevant only to the development of a prediction model; E=items relating solely to the evaluation of a prediction model; D;E=items applicable to both the development and evaluation of a prediction model

^2^ Separately for all model building approaches.

^3^ TRIPOD-Cluster is a checklist of reporting recommendations for studies developing or validating models that explicitly account for clustering or explore heterogeneity in model performance (eg, at different hospitals or centres). Debray et al, BMJ 2023; 380: e071018 [DOI: 10.1136/bmj-2022-071018]

| *Training versus*  *evaluation* | 16 | D;E | Identify any differences between the development and evaluation data in healthcare setting, eligibility  criteria, outcome, and predictors | 11 |
| --- | --- | --- | --- | --- |
| *Ethical approval* | 17 | D;E | Name the institutional research board or ethics committee that approved the study and describe the participant-informed consent or the ethics committee waiver of informed consent | 26 |
| **OPEN SCIENCE** | | | | |
| *Funding* | 18a | D;E | Give the source of funding and the role of the funders for the present study | 25 |
| *Conflicts of interest* | 18b | D;E | Declare any conflicts of interest and financial disclosures for all authors | 26 |
| *Protocol* | 18c | D;E | Indicate where the study protocol can be accessed or state that a protocol was not prepared | 26 |
| *Registration* | 18d | D;E | Provide registration information for the study, including register name and registration number, or state  that the study was not registered | 26 |
| *Data sharing* | 18e | D;E | Provide details of the availability of the study data | 25 |
| *Code sharing* | 18f | D;E | Provide details of the availability of the analytical code^4^ | 25 |
| **PATIENT & PUBLIC INVOLVEMENT** | | | | |
| *Patient & Public Involvement* | 19 | D;E | Provide details of any patient and public involvement during the design, conduct, reporting, interpretation, or dissemination of the study or state no involvement. | 5 |
| **RESULTS** | | | | |
| *Participants* | 20a | D;E | Describe the flow of participants through the study, including the number of participants with and without the outcome and, if applicable, a summary of the follow-up time. A diagram may be helpful. | 10 |
|  | 20b | D;E | Report the characteristics overall and, where applicable, for each data source or setting, including the key dates, key predictors (including demographics), treatments received, sample size, number of outcome events, follow-up time, and amount of missing data. A table may be helpful. Report any  differences across key demographic groups. | 11 |
|  | 20c | E | For model evaluation, show a comparison with the development data of the distribution of important predictors (demographics, predictors, and outcome). | 11 |
| *Model development* | 21 | D;E | Specify the number of participants and outcome events in each analysis (e.g., for model development, hyperparameter tuning, model evaluation) | 12 |
| *Model specification* | 22 | D | Provide details of the full prediction model (e.g., formula, code, object, application programming interface) to allow predictions in new individuals and to enable third-party evaluation and implementation, including any restrictions to access or re-use (e.g., freely available, proprietary)^5^ | 14 |
| *Model performance* | 23a | D;E | Report model performance estimates with confidence intervals, including for any key subgroups (e.g., sociodemographic). Consider plots to aid presentation. | 17 |
|  | 23b | D;E | If examined, report results of any heterogeneity in model performance across clusters. See TRIPOD  Cluster for additional details^3^. | NA |
| *Model updating* | 24 | E | Report the results from any model updating, including the updated model and subsequent performance | 17 |
| **DISCUSSION** | | | | |
| *Interpretation* | 25 | D;E | Give an overall interpretation of the main results, including issues of fairness in the context of the  objectives and previous studies | 22 |
| *Limitations* | 26 | D;E | Discuss any limitations of the study (such as a non-representative sample, sample size, overfitting, missing data) and their effects on any biases, statistical uncertainty, and generalizability | 23 |
| *Usability of the model in the context of current care* | 27a | D | Describe how poor quality or unavailable input data (e.g., predictor values) should be assessed and handled when implementing the prediction model | 24-25 |
|  | 27b | D | Specify whether users will be required to interact in the handling of the input data or use of the model,  and what level of expertise is required of users | NA |
|  | 27c | D;E | Discuss any next steps for future research, with a specific view to applicability and generalizability of  the model | 24 |

From: Collins GS, Moons KGM, Dhiman P, et al. *BMJ* 2024;385:e078378. doi:10.1136/bmj-2023-078378

^4^ This relates to the analysis code, for example, any data cleaning, feature engineering, model building, evaluation.

^5^ This relates to the code to implement the model to get estimates of risk for a new individu

## Table 4: Hazard ratios from sensitivity analysis based on complete case analysis (CCA)

| **Predictors** | **Model 1 HR (95% CI)** | **Model 2a HR (95% CI)** | **Model 2b HR (95% CI)** |
| --- | --- | --- | --- |
| QRISK3 linear predictor 1 | 1.08 (1.06 to 1.09) | - | - |
| QRISK3 linear predictor 2 | 0.97 (0.96 to 0.98) | - | - |
| Age1 | - | 2.37 (2.13 to 2.63) | 2.28 (2.04 to 2.55) |
| BMI 1 | - | 1.48 (1.35 to 1.62) | 1.43 (1.30 to 1.57) |
| SBP 1 | - | 2.03 (1.72 to 2.39) | 1.97 (1.67 to 2.32) |
| Townsend scores | - | 1.13 (1.08 to 1.19) | 1.14 (1.08 to 1.19) |
| Family history of CVD | - | 1.60 (1.27 to 2.03) | 1.58 (1.25 to 2.00) |
| Black ethnicity | - | 1.56 (1.30 to 1.86) | 1.56 (1.30 to 1.86) |
| Mixed ethnicity | - | 0.94 (0.49 to 1.82) | 0.96 (0.50 to 1.86) |
| Asian ethnicity | - | 1.36 (1.03 to 1.79) | 1.33 (1.01 to 1.76) |
| Other ethnicity | - | 1.02 (0.76 to 1.36) | 1.00 (0.75 to 1.34) |
| Ex smoker | - | 1.11 (0.93 to 1.33) | 1.09 (0.92 to 1.31) |
| Current smoker | - | 2.26 (1.96 to 2.60) | 2.22 (1.93 to 2.55) |
| Atrial Fibrillation | - | 1.99 (0.62 to 6.36) | 2.12 (0.66 to 6.75) |
| Migraine | - | 1.83 (1.56 to 2.15) | 1.73 (1.47 to 2.03) |
| Rhematoid Arthritis | - | 1.02 (0.44 to 2.34) | 1.02 (0.45 to 2.35) |
| CKD | - | 4.02 (1.78 to 9.08) | 3.73 (1.65 to 8.47) |
| Severe mental illness | - | 1.84 (1.24 to 2.73) | 1.77 (1.19 to 2.62) |
| SLE | - | 4.56 (2.47 to 8.41) | 4.13 (2.24 to 7.62) |
| Diabetes Type 1 | - | 5.39 (3.27 to 8.87) | 5.06 (3.06 to 8.37) |
| Diabetes Type 2 | - | 4.03 (2.91 to 5.59) | 3.68 (2.61 to 5.18) |
| Atypical antipsychotics | - | 1.18 (0.57 to 2.44) | 1.15 (0.56 to 2.38) |
| Corticosteroid use | - | 2.01 (1.19 to 3.40) | 1.85 (1.09 to 3.13) |
| Treated hypertension | - | 1.52 (1.05 to 2.21) | 1.44 (0.99 to 2.09) |
| Pre-eclampsia | 1.45 (1.14 to 1.84) | - | 1.49 (1.17 to 1.90) |
| Postnatal depression | 1.32 (1.07 to 1.64) | - | 1.28 (1.04 to 1.59) |
| GDM | 1.12 (0.86 to 1.45) | - | 1.18 (0.89 to 1.54) |
| Gestational hypertension | 1.23 (0.93 to 1.62) | - | 1.22 (0.92 to 1.61) |
| Miscarriage | 1.02 (0.88 to 1.17) | - | 1.06 (0.92 to 1.22) |
| Preterm birth | 1.34 (1.07 to 1.68) | - | - |
| Stillbirth | 1.13 (0.73 to 1.75) | - | 1.14 (0.74 to 1.77) |
| Small for gestational age | 1.00 (0.76 to 1.31) | - | - |
| Number of pregnancies | 1.06 (1.03 to 1.09) | - | 1.06 (1.03 to 1.09) |
| Endometriosis | 1.68 (1.27 to 2.22) | - | 1.73 (1.30 to 2.28) |
| PCOS | 1.33 (1.02 to 1.74) | - | 1.34 (1.03 to 1.75) |
| Irregular menses | 1.20 (1.03 to 1.40) | - | - |
| Placental abruption | 1.15 (0.59 to 2.22) | - | - |

## Table 5: Hazard ratios from sensitivity analysis restricting the data to those aged 25 years of age and above

| **Predictors** | **Model 1 HR (95% CI)** | **Model 2a HR (95% CI)** | **Model 2b HR (95% CI)** |
| --- | --- | --- | --- |
| QRISK3 linear predictor 1 | 1.06 (1.05 to 1.07) | - | - |
| QRISK3 linear predictor 2 | 0.98 (0.97 to 0.98) | - | - |
| Age1 | - | 2.16 (1.96 to 2.37) | 2.06 (1.87 to 2.28) |
| BMI 1 | - | 1.36 (1.25 to 1.48) | 1.30 (1.19 to 1.42) |
| SBP 1 | - | 1.95 (1.69 to 2.25) | 1.88 (1.62 to 2.17) |
| Townsend scores | - | 1.19 (1.14 to 1.23) | 1.19 (1.14 to 1.23) |
| Family history of CVD | - | 1.55 (1.25 to 1.93) | 1.52 (1.22 to 1.89) |
| Black ethnicity | - | 1.55 (1.34 to 1.80) | 1.56 (1.35 to 1.81) |
| Mixed ethnicity | - | 1.33 (0.85 to 2.07) | 1.36 (0.87 to 2.12) |
| Asian ethnicity | - | 1.23 (0.98 to 1.53) | 1.21 (0.97 to 1.51) |
| Other ethnicity | - | 0.91 (0.72 to 1.16) | 0.92 (0.72 to 1.17) |
| Ex smoker | - | 0.91 (0.78 to 1.07) | 0.89 (0.75 to 1.04) |
| Current smoker | - | 1.83 (1.63 to 2.06) | 1.77 (1.57 to 1.99) |
| Atrial Fibrillation | - | 1.65 (0.52 to 5.20) | 1.75 (0.55 to 5.52) |
| Migraine | - | 1.75 (1.52 to 2.01) | 1.63 (1.41 to 1.87) |
| Rhematoid Arthritis | - | 1.12 (0.55 to 2.30) | 1.10 (0.54 to 2.24) |
| CKD | - | 4.46 (2.20 to 9.04) | 4.12 (2.02 to 8.39) |
| Severe mental illness | - | 2.04 (1.45 to 2.87) | 1.93 (1.38 to 2.71) |
| SLE | - | 3.72 (2.08 to 6.67) | 3.35 (1.87 to 5.99) |
| Diabetes Type 1 | - | 5.39 (3.52 to 8.24) | 4.87 (3.17 to 7.49) |
| Diabetes Type 2 | - | 4.41 (3.35 to 5.80) | 3.90 (2.92 to 5.20) |
| Atypical antipsychotics | - | 1.00 (0.49 to 2.06) | 0.99 (0.48 to 2.02) |
| Corticosteroid use | - | 1.95 (1.18 to 3.24) | 1.77 (1.07 to 2.94) |
| Treated hypertension | - | 1.54 (1.09 to 2.18) | 1.43 (1.01 to 2.03) |
| Pre-eclampsia | 1.54 (1.25 to 1.88) | - | 1.53 (1.25 to 1.88) |
| Postnatal depression | 1.38 (1.16 to 1.65) | - | 1.35 (1.12 to 1.61) |
| GDM | 1.22 (0.98 to 1.52) | - | 1.20 (0.96 to 1.51) |
| Gestational hypertension | 1.34 (1.06 to 1.69) | - | 1.32 (1.05 to 1.67) |
| Miscarriage | 1.09 (0.97 to 1.22) | - | 1.13 (1.00 to 1.27) |
| Preterm birth | 1.17 (0.94 to 1.45) | - | - |
| Stillbirth | 1.44 (1.03 to 2.00) | - | 1.40 (1.01 to 1.95) |
| Small for gestational age | 0.94 (0.74 to 1.19) | - | - |
| Number of pregnancies | 1.07 (1.05 to 1.10) | - | 1.07 (1.05 to 1.10) |
| Endometriosis | 1.50 (1.17 to 1.91) | - | 1.56 (1.22 to 1.99) |
| PCOS | 1.44 (1.15 to 1.79) | - | 1.42 (1.14 to 1.77) |
| Irregular menses | 1.03 (0.90 to 1.19) | - | - |
| Placental abruption | 1.28 (0.75 to 2.17) | - | - |

## Table 6: Hazard ratios from sensitivity analysis using multiple imputation for missing data

| **Predictors** | **Model 1 HR (95% CI)** | **Model 2a HR (95% CI)** | **Model 2b HR (95% CI)** |
| --- | --- | --- | --- |
| QRISK3 linear predictor 1 | 1.06 (1.05 to 1.07) | - | - |
| QRISK3 linear predictor 2 | 0.98 (0.97 to 0.98) | - | - |
| Age1 | - | 2.21 (2.03 to 2.40) | 2.07 (1.89 to 2.25) |
| BMI 1 | - | 1.33 (1.22 to 1.45) | 1.26 (1.16 to 1.38) |
| SBP 1 | - | 1.87 (1.62 to 2.15) | 1.78 (1.54 to 2.05) |
| Townsend scores | - | 1.18 (1.14 to 1.23) | 1.18 (1.14 to 1.23) |
| Family history of CVD | - | 1.56 (1.25 to 1.95) | 1.51 (1.21 to 1.89) |
| Black ethnicity | - | 1.54 (1.33 to 1.79) | 1.55 (1.34 to 1.79) |
| Mixed ethnicity | - | 1.21 (0.78 to 1.88) | 1.24 (0.80 to 1.94) |
| Asian ethnicity | - | 1.25 (1.00 to 1.56) | 1.23 (0.99 to 1.54) |
| Other ethnicity | - | 0.90 (0.70 to 1.15) | 0.91 (0.71 to 1.16) |
| Ex smoker | - | 1.09 (0.83 to 1.43) | 1.07 (0.81 to 1.43) |
| Current smoker | - | 1.86 (1.61 to 2.15) | 1.82 (1.58 to 2.10) |
| Atrial Fibrillation | - | 2.73 (1.11 to 6.72) | 2.73 (1.10 to 6.77) |
| Migraine | - | 1.72 (1.50 to 1.98) | 1.60 (1.39 to 1.84) |
| Rhematoid Arthritis | - | 1.07 (0.52 to 2.21) | 1.03 (0.50 to 2.12) |
| CKD | - | 4.32 (2.13 to 8.77) | 3.81 (1.87 to 7.76) |
| Severe mental illness | - | 2.02 (1.43 to 2.83) | 1.88 (1.34 to 2.64) |
| SLE | - | 3.96 (2.25 to 6.97) | 3.50 (1.99 to 6.18) |
| Diabetes Type 1 | - | 5.75 (3.86 to 8.57) | 5.25 (3.50 to 7.86) |
| Diabetes Type 2 | - | 4.39 (3.34 to 5.78) | 3.87 (2.90 to 5.16) |
| Atypical antipsychotics | - | 1.02 (0.50 to 2.10) | 1.00 (0.49 to 2.05) |
| Corticosteroid use | - | 1.95 (1.17 to 3.25) | 1.72 (1.03 to 2.86) |
| Treated hypertension | - | 1.61 (1.11 to 2.33) | 1.40 (0.96 to 2.03) |
| Pre-eclampsia | 1.50 (1.23 to 1.84) | - | 1.55 (1.26 to 1.90) |
| Postnatal depression | 1.34 (1.12 to 1.60) | - | 1.32 (1.11 to 1.58) |
| GDM | 1.17 (0.94 to 1.46) | - | 1.16 (0.93 to 1.46) |
| Gestational hypertension | 1.54 (1.28 to 1.86) | - | 1.52 (1.26 to 1.85) |
| Miscarriage | 1.11 (0.99 to 1.25) | - | 1.14 (1.02 to 1.28) |
| Preterm birth | 1.21 (0.98 to 1.49) | - | - |
| Stillbirth | 1.38 (0.99 to 1.93) | - | 1.36 (0.98 to 1.89) |
| Small for gestational age | 0.91 (0.72 to 1.14) | - | - |
| Number of pregnancies | 1.07 (1.05 to 1.10) | - | 1.08 (1.05 to 1.10) |
| Endometriosis | 1.51 (1.19 to 1.93) | - | 1.56 (1.22 to 1.99) |
| PCOS | 1.39 (1.11 to 1.72) | - | 1.40 (1.13 to 1.74) |
| Irregular menses | 1.07 (0.93 to 1.24) | - | - |
| Placental abruption | 1.25 (0.73 to 2.12) | - | - |

## Table 7: Discrimination by sub-groups in main analysis

### Table 7a: Age groups

| **Model** | **Sub-group** | **Number of women (%)** | **CVD Events within 10 years** | **Harrell's C** | **Royston's D** | **R2D** | **Distribution of LP** |
| --- | --- | --- | --- | --- | --- | --- | --- |
| QRISK@3 | <25 | 97121 (17.1) | 73 | 0.570 (0.484 to 0.655) | 0.616 (0.241 to 0.991) | 0.0831 | -2.347 (0.482) |
| Model 1 | <25 | 97121 (17.1) | 73 | 0.599 (0.518 to 0.681) | 0.782 (0.414 to 1.151) | 0.1275 | -0.681 (0.293) |
| Model 2a | <25 | 97121 (17.1) | 73 | 0.564 (0.477 to 0.651) | 0.511 (0.148 to 0.875) | 0.0588 | -0.523 (0.537) |
| Model 2b | <25 | 97121 (17.1) | 73 | 0.577 (0.490 to 0.664) | 0.577 (0.215 to 0.939) | 0.0735 | -0.475 (0.562) |
| QRISK@3 | 15-34 | 369539 (65.1) | 599 | 0.627 (0.600 to 0.654) | 0.842 (0.710 to 0.974) | 0.1447 | -1.604 (0.751) |
| Model 1 | 15-34 | 369539 (65.1) | 599 | 0.653 (0.626 to 0.680) | 1.005 (0.872 to 1.138) | 0.1943 | -0.244 (0.535) |
| Model 2a | 15-34 | 369539 (65.1) | 599 | 0.725 (0.708 to 0.741) | 1.406 (1.307 to 1.505) | 0.3205 | 0.745 (0.637) |
| Model 2b | 15-34 | 369539 (65.1) | 599 | 0.658 (0.631 to 0.684) | 1.038 (0.908 to 1.168) | 0.2046 | 0.021 (0.665) |
| QRISK@3 | 25-34 | 272418 (48.0) | 526 | 0.597 (0.567 to 0.628) | 0.697 (0.556 to 0.838) | 0.1039 | -1.339 (0.644) |
| Model 1 | 25-34 | 272418 (48.0) | 526 | 0.630 (0.600 to 0.660) | 0.902 (0.760 to 1.044) | 0.1627 | -0.088 (0.515) |
| Model 2a | 25-34 | 272418 (48.0) | 526 | 0.619 (0.589 to 0.648) | 0.833 (0.696 to 0.970) | 0.1421 | 0.106 (0.554) |
| Model 2b | 25-34 | 272418 (48.0) | 526 | 0.642 (0.613 to 0.671) | 0.985 (0.847 to 1.124) | 0.1882 | 0.197 (0.607) |
| QRISK@3 | 35-49 | 198128 (34.9) | 1014 | 0.706 (0.688 to 0.724) | 1.318 (1.219 to 1.418) | 0.2933 | -0.227 (0.570) |
| Model 1 | 35-49 | 198128 (34.9) | 1014 | 0.714 (0.695 to 0.732) | 1.396 (1.295 to 1.496) | 0.3174 | 0.770 (0.610) |
| Model 2a | 35-49 | 198128 (34.9) | 1014 | 0.638 (0.611 to 0.665) | 0.913 (0.784 to 1.042) | 0.1659 | -0.059 (0.615) |
| Model 2b | 35-49 | 198128 (34.9) | 1014 | 0.729 (0.712 to 0.747) | 1.476 (1.376 to 1.577) | 0.3422 | 0.849 (0.687) |

### Table 7b: Ethnicity

| **Model** | **Sub-group** | **Number of women (%)** | **CVD Events within 10 years** | **Harrell's C** | **Royston's D** | **R2D** | **Distribution of LP** |
| --- | --- | --- | --- | --- | --- | --- | --- |
| QRISK@3 | Asian | 35390 ( 6.2) | 92 | 0.817 (0.766 to 0.868) | 1.927 (1.586 to 2.268) | 0.4699 | -0.927 (0.913) |
| Model 1 | Asian | 35390 ( 6.2) | 92 | 0.837 (0.789 to 0.885) | 2.050 (1.705 to 2.395) | 0.5008 | 0.222 (0.773) |
| Model 2a | Asian | 35390 ( 6.2) | 92 | 0.820 (0.771 to 0.868) | 1.859 (1.529 to 2.189) | 0.452 | 0.342 (0.651) |
| Model 2b | Asian | 35390 ( 6.2) | 92 | 0.836 (0.788 to 0.883) | 1.977 (1.644 to 2.310) | 0.4827 | 0.418 (0.711) |
| QRISK@3 | Black | 42547 ( 7.5) | 239 | 0.725 (0.690 to 0.761) | 1.368 (1.150 to 1.586) | 0.3088 | -1.126 (1.031) |
| Model 1 | Black | 42547 ( 7.5) | 239 | 0.737 (0.701 to 0.772) | 1.465 (1.245 to 1.684) | 0.3387 | 0.139 (0.796) |
| Model 2a | Black | 42547 ( 7.5) | 239 | 0.715 (0.679 to 0.750) | 1.263 (1.053 to 1.474) | 0.2759 | 0.840 (0.769) |
| Model 2b | Black | 42547 ( 7.5) | 239 | 0.726 (0.689 to 0.762) | 1.366 (1.153 to 1.579) | 0.3081 | 0.927 (0.817) |
| QRISK@3 | Mixed | 8026 ( 1.4) | 20 | 0.628 (0.492 to 0.765) | 1.613 (0.891 to 2.335) | 0.3832 | -1.337 (0.983) |
| Model 1 | Mixed | 8026 ( 1.4) | 20 | 0.697 (0.575 to 0.820) | 1.766 (1.041 to 2.492) | 0.4269 | -0.048 (0.726) |
| Model 2a | Mixed | 8026 ( 1.4) | 20 | 0.657 (0.527 to 0.786) | 1.703 (1.002 to 2.404) | 0.4091 | 0.315 (0.724) |
| Model 2b | Mixed | 8026 ( 1.4) | 20 | 0.716 (0.590 to 0.842) | 1.903 (1.188 to 2.618) | 0.4636 | 0.406 (0.767) |
| QRISK@3 | Others | 28451 ( 5.0) | 71 | 0.743 (0.675 to 0.810) | 1.494 (1.102 to 1.885) | 0.3476 | -1.156 (0.938) |
| Model 1 | Others | 28451 ( 5.0) | 71 | 0.752 (0.686 to 0.819) | 1.610 (1.219 to 2.001) | 0.3822 | 0.057 (0.716) |
| Model 2a | Others | 28451 ( 5.0) | 71 | 0.757 (0.694 to 0.821) | 1.555 (1.172 to 1.937) | 0.3659 | 0.151 (0.683) |
| Model 2b | Others | 28451 ( 5.0) | 71 | 0.770 (0.708 to 0.831) | 1.653 (1.273 to 2.034) | 0.3949 | 0.220 (0.729) |
| QRISK@3 | White | 434601 (76.6) | 1176 | 0.689 (0.671 to 0.707) | 1.292 (1.196 to 1.388) | 0.2851 | -1.128 (0.948) |
| Model 1 | White | 434601 (76.6) | 1176 | 0.700 (0.682 to 0.718) | 1.383 (1.286 to 1.480) | 0.3136 | 0.112 (0.735) |
| Model 2a | White | 434601 (76.6) | 1176 | 0.699 (0.681 to 0.717) | 1.326 (1.233 to 1.420) | 0.2957 | 0.161 (0.708) |
| Model 2b | White | 434601 (76.6) | 1176 | 0.707 (0.690 to 0.725) | 1.405 (1.310 to 1.500) | 0.3204 | 0.255 (0.759) |
| QRISK@3 | Unknown | 18652 ( 3.3) | 15 | 0.670 (0.518 to 0.821) | 0.797 (-0.026 to 1.620) | 0.1316 | -1.248 (0.964) |
| Model 1 | Unknown | 18652 ( 3.3) | 15 | 0.659 (0.506 to 0.812) | 0.997 (0.168 to 1.825) | 0.1917 | -0.070 (0.684) |
| Model 2a | Unknown | 18652 ( 3.3) | 15 | 0.668 (0.509 to 0.826) | 0.756 (-0.047 to 1.559) | 0.1201 | 0.057 (0.696) |
| Model 2b | Unknown | 18652 ( 3.3) | 15 | 0.656 (0.498 to 0.814) | 0.793 (-0.013 to 1.599) | 0.1305 | 0.054 (0.705) |

### Table 7c: Reproductive and pregnancy complications

| **Model** | **Sub-group** | **Number of women (%)** | **CVD Events within 10 years** | **Harrell's C** | **Royston's D** | **R2D** | **Distribution of LP** |
| --- | --- | --- | --- | --- | --- | --- | --- |
| QRISK@3 | Endometriosis | 12515 ( 2.2) | 68 | 0.648 (0.573 to 0.722) | 1.009 (0.617 to 1.401) | 0.1955 | -0.723 (0.843) |
| Model 1 | Endometriosis | 12515 ( 2.2) | 68 | 0.680 (0.613 to 0.747) | 1.175 (0.779 to 1.572) | 0.2479 | 0.820 (0.714) |
| Model 2a | Endometriosis | 12515 ( 2.2) | 68 | 0.679 (0.605 to 0.754) | 1.186 (0.801 to 1.571) | 0.2514 | 0.438 (0.695) |
| Model 2b | Endometriosis | 12515 ( 2.2) | 68 | 0.701 (0.631 to 0.771) | 1.299 (0.907 to 1.691) | 0.2872 | 1.000 (0.739) |
| QRISK@3 | GDM | 16257 ( 2.9) | 92 | 0.739 (0.679 to 0.800) | 1.504 (1.167 to 1.841) | 0.3506 | -0.477 (0.951) |
| Model 1 | GDM | 16257 ( 2.9) | 92 | 0.761 (0.703 to 0.819) | 1.601 (1.265 to 1.937) | 0.3796 | 0.859 (0.878) |
| Model 2a | GDM | 16257 ( 2.9) | 92 | 0.739 (0.676 to 0.801) | 1.447 (1.117 to 1.777) | 0.3333 | 0.754 (0.849) |
| Model 2b | GDM | 16257 ( 2.9) | 92 | 0.757 (0.694 to 0.821) | 1.557 (1.225 to 1.890) | 0.3667 | 1.057 (0.894) |
| QRISK@3 | Gestational hypertension | 20052 ( 3.5) | 122 | 0.752 (0.699 to 0.805) | 1.678 (1.379 to 1.976) | 0.4019 | -0.726 (0.974) |
| Model 1 | Gestational hypertension | 20052 ( 3.5) | 122 | 0.764 (0.714 to 0.814) | 1.732 (1.436 to 2.028) | 0.4174 | 0.858 (0.819) |
| Model 2a | Gestational hypertension | 20052 ( 3.5) | 122 | 0.779 (0.733 to 0.825) | 1.742 (1.457 to 2.028) | 0.4202 | 0.548 (0.818) |
| Model 2b | Gestational hypertension | 20052 ( 3.5) | 122 | 0.784 (0.740 to 0.829) | 1.800 (1.515 to 2.085) | 0.4361 | 1.044 (0.839) |
| QRISK@3 | Irregular menses | 67587 (11.9) | 247 | 0.704 (0.662 to 0.746) | 1.438 (1.227 to 1.648) | 0.3304 | -0.952 (0.974) |
| Model 1 | Irregular menses | 67587 (11.9) | 247 | 0.719 (0.679 to 0.760) | 1.533 (1.321 to 1.746) | 0.3595 | 0.366 (0.811) |
| Model 2a | Irregular menses | 67587 (11.9) | 247 | 0.717 (0.676 to 0.757) | 1.508 (1.302 to 1.714) | 0.3519 | 0.385 (0.781) |
| Model 2b | Irregular menses | 67587 (11.9) | 247 | 0.728 (0.689 to 0.768) | 1.572 (1.362 to 1.782) | 0.3711 | 0.553 (0.845) |
| QRISK@3 | Miscarriage | 127020 (22.4) | 563 | 0.691 (0.665 to 0.717) | 1.263 (1.124 to 1.402) | 0.2758 | -0.786 (0.943) |
| Model 1 | Miscarriage | 127020 (22.4) | 563 | 0.706 (0.681 to 0.732) | 1.375 (1.235 to 1.515) | 0.311 | 0.534 (0.773) |
| Model 2a | Miscarriage | 127020 (22.4) | 563 | 0.711 (0.686 to 0.736) | 1.363 (1.227 to 1.498) | 0.3071 | 0.446 (0.748) |
| Model 2b | Miscarriage | 127020 (22.4) | 563 | 0.720 (0.695 to 0.745) | 1.456 (1.319 to 1.593) | 0.3361 | 0.737 (0.786) |
| QRISK@3 | PCOS | 21306 ( 3.8) | 88 | 0.711 (0.641 to 0.781) | 1.274 (0.922 to 1.626) | 0.2794 | -0.863 (0.892) |
| Model 1 | PCOS | 21306 ( 3.8) | 88 | 0.719 (0.653 to 0.786) | 1.313 (0.960 to 1.667) | 0.2917 | 0.675 (0.763) |
| Model 2a | PCOS | 21306 ( 3.8) | 88 | 0.736 (0.677 to 0.796) | 1.457 (1.114 to 1.799) | 0.3362 | 0.417 (0.741) |
| Model 2b | PCOS | 21306 ( 3.8) | 88 | 0.741 (0.683 to 0.800) | 1.504 (1.157 to 1.850) | 0.3505 | 0.868 (0.791) |
| QRISK@3 | Placental abruption | 2954 ( 0.5) | 14 | 0.684 (0.510 to 0.859) | 0.879 (0.033 to 1.726) | 0.1559 | -0.887 (0.929) |
| Model 1 | Placental abruption | 2954 ( 0.5) | 14 | 0.670 (0.497 to 0.843) | 0.860 (0.011 to 1.709) | 0.1499 | 0.671 (0.812) |
| Model 2a | Placental abruption | 2954 ( 0.5) | 14 | 0.699 (0.530 to 0.868) | 1.004 (0.183 to 1.826) | 0.1941 | 0.421 (0.736) |
| Model 2b | Placental abruption | 2954 ( 0.5) | 14 | 0.687 (0.521 to 0.852) | 0.919 (0.090 to 1.748) | 0.1679 | 0.660 (0.831) |
| QRISK@3 | Postpartum depression | 30732 ( 5.4) | 138 | 0.714 (0.664 to 0.763) | 1.500 (1.219 to 1.781) | 0.3494 | -1.012 (0.943) |
| Model 1 | Postpartum depression | 30732 ( 5.4) | 138 | 0.712 (0.664 to 0.761) | 1.511 (1.228 to 1.793) | 0.3527 | 0.568 (0.774) |
| Model 2a | Postpartum depression | 30732 ( 5.4) | 138 | 0.727 (0.676 to 0.778) | 1.541 (1.268 to 1.813) | 0.3617 | 0.340 (0.740) |
| Model 2b | Postpartum depression | 30732 ( 5.4) | 138 | 0.729 (0.679 to 0.778) | 1.574 (1.298 to 1.849) | 0.3715 | 0.774 (0.804) |
| QRISK@3 | Preeclampsia | 18676 ( 3.3) | 107 | 0.767 (0.716 to 0.818) | 1.656 (1.342 to 1.971) | 0.3957 | -0.814 (0.956) |
| Model 1 | Preeclampsia | 18676 ( 3.3) | 107 | 0.775 (0.725 to 0.824) | 1.741 (1.426 to 2.056) | 0.4199 | 0.793 (0.789) |
| Model 2a | Preeclampsia | 18676 ( 3.3) | 107 | 0.779 (0.732 to 0.826) | 1.658 (1.351 to 1.964) | 0.3962 | 0.485 (0.783) |
| Model 2b | Preeclampsia | 18676 ( 3.3) | 107 | 0.785 (0.737 to 0.833) | 1.753 (1.441 to 2.064) | 0.423 | 1.005 (0.807) |
| QRISK@3 | Preterm birth | 23525 ( 4.1) | 99 | 0.765 (0.705 to 0.825) | 1.905 (1.573 to 2.237) | 0.4642 | -0.914 (0.961) |
| Model 1 | Preterm birth | 23525 ( 4.1) | 99 | 0.770 (0.711 to 0.829) | 1.912 (1.580 to 2.244) | 0.466 | 0.539 (0.830) |
| Model 2a | Preterm birth | 23525 ( 4.1) | 99 | 0.768 (0.706 to 0.830) | 1.907 (1.584 to 2.230) | 0.4647 | 0.396 (0.777) |
| Model 2b | Preterm birth | 23525 ( 4.1) | 99 | 0.771 (0.710 to 0.832) | 1.931 (1.603 to 2.259) | 0.4711 | 0.598 (0.863) |
| QRISK@3 | SGA | 34935 ( 6.2) | 74 | 0.679 (0.602 to 0.756) | 1.324 (0.941 to 1.708) | 0.2951 | -1.183 (0.920) |
| Model 1 | SGA | 34935 ( 6.2) | 74 | 0.702 (0.628 to 0.775) | 1.503 (1.119 to 1.887) | 0.3505 | 0.011 (0.730) |
| Model 2a | SGA | 34935 ( 6.2) | 74 | 0.666 (0.588 to 0.745) | 1.376 (1.000 to 1.751) | 0.3112 | 0.194 (0.701) |
| Model 2b | SGA | 34935 ( 6.2) | 74 | 0.689 (0.611 to 0.768) | 1.558 (1.179 to 1.937) | 0.3669 | 0.321 (0.771) |
| QRISK@3 | Stillbirth | 5254 ( 0.9) | 37 | 0.695 (0.583 to 0.806) | 1.240 (0.697 to 1.782) | 0.2684 | -0.727 (0.965) |
| Model 1 | Stillbirth | 5254 ( 0.9) | 37 | 0.711 (0.607 to 0.815) | 1.308 (0.770 to 1.847) | 0.2901 | 0.927 (0.851) |
| Model 2a | Stillbirth | 5254 ( 0.9) | 37 | 0.713 (0.606 to 0.821) | 1.254 (0.728 to 1.781) | 0.273 | 0.562 (0.797) |
| Model 2b | Stillbirth | 5254 ( 0.9) | 37 | 0.730 (0.630 to 0.830) | 1.335 (0.802 to 1.868) | 0.2985 | 1.120 (0.867) |

## Table 8: Overall discrimination in sensitivity analysis based on complete case analysis

| Model | Harrell's C | Royston's D | R2D | Distribution of LP |
| --- | --- | --- | --- | --- |
| QRISK3 | 0.723 (0.704 to 0.742) | 1.537 (1.435 to 1.640) | 0.3607 | -0.998 (0.933) |
| Model 1 | 0.733 (0.714 to 0.752) | 1.630 (1.527 to 1.733) | 0.388 | 0.124 (0.836) |
| Model 2a | 0.738 (0.719 to 0.756) | 1.583 (1.484 to 1.683) | 0.3744 | 0.372 (0.808) |
| Model 2b | 0.742 (0.724 to 0.761) | 1.652 (1.551 to 1.753) | 0.3944 | 0.449 (0.857) |

### Table 9a: Discrimination by Age groups in complete case analysis

| **Model** | **Sub-group** | **Number of women (%)** | **CVD Events within 10 years** | **Harrell's C** | **Royston's D** | **R2D** | **Distribution of LP** |
| --- | --- | --- | --- | --- | --- | --- | --- |
| QRISK@3 | <25 | 54068 (13.7) | 29 | 0.588 (0.429 to 0.746) | 0.672 (0.088 to 1.256) | 0.0973 | -2.244 (0.539) |
| Model 1 | <25 | 54068 (13.7) | 29 | 0.616 (0.470 to 0.762) | 0.726 (0.142 to 1.310) | 0.1117 | -0.817 (0.359) |
| Model 2a | <25 | 54068 (13.7) | 29 | 0.594 (0.433 to 0.755) | 0.687 (0.118 to 1.257) | 0.1014 | -0.435 (0.631) |
| Model 2b | <25 | 54068 (13.7) | 29 | 0.591 (0.430 to 0.751) | 0.683 (0.113 to 1.252) | 0.1001 | -0.414 (0.651) |
| QRISK@3 | 15-34 | 248309 (62.8) | 351 | 0.649 (0.613 to 0.686) | 1.005 (0.831 to 1.180) | 0.1944 | -1.483 (0.750) |
| Model 1 | 15-34 | 248309 (62.8) | 351 | 0.667 (0.632 to 0.703) | 1.133 (0.957 to 1.309) | 0.2347 | -0.292 (0.614) |
| Model 2a | 15-34 | 248309 (62.8) | 351 | 0.744 (0.724 to 0.764) | 1.533 (1.413 to 1.653) | 0.3593 | 0.890 (0.719) |
| Model 2b | 15-34 | 248309 (62.8) | 351 | 0.679 (0.643 to 0.714) | 1.190 (1.018 to 1.361) | 0.2525 | 0.114 (0.725) |
| QRISK@3 | 25-34 | 194241 (49.1) | 322 | 0.630 (0.590 to 0.669) | 0.896 (0.715 to 1.077) | 0.1608 | -1.272 (0.657) |
| Model 1 | 25-34 | 194241 (49.1) | 322 | 0.650 (0.612 to 0.689) | 1.050 (0.867 to 1.232) | 0.2082 | -0.145 (0.590) |
| Model 2a | 25-34 | 194241 (49.1) | 322 | 0.654 (0.616 to 0.691) | 1.033 (0.857 to 1.208) | 0.203 | 0.191 (0.633) |
| Model 2b | 25-34 | 194241 (49.1) | 322 | 0.667 (0.630 to 0.704) | 1.135 (0.957 to 1.313) | 0.2352 | 0.261 (0.675) |
| QRISK@3 | 35-49 | 147317 (37.2) | 696 | 0.739 (0.718 to 0.759) | 1.502 (1.382 to 1.623) | 0.3501 | -0.187 (0.592) |
| Model 1 | 35-49 | 147317 (37.2) | 696 | 0.743 (0.723 to 0.764) | 1.566 (1.445 to 1.688) | 0.3694 | 0.816 (0.678) |
| Model 2a | 35-49 | 147317 (37.2) | 696 | 0.666 (0.630 to 0.702) | 1.100 (0.931 to 1.269) | 0.2242 | 0.055 (0.683) |
| Model 2b | 35-49 | 147317 (37.2) | 696 | 0.747 (0.726 to 0.768) | 1.590 (1.469 to 1.711) | 0.3763 | 0.983 (0.755) |

### Table 9b: Discrimination by Ethnicity in complete case analysis

| **Model** | **Sub-group** | **Number of women (%)** | **CVD Events within 10 years** | **Harrell's C** | **Royston's D** | **R2D** | **Distribution of LP** |
| --- | --- | --- | --- | --- | --- | --- | --- |
| QRISK@3 | Asian | 25317 ( 6.4) | 57 | 0.801 (0.736 to 0.865) | 1.743 (1.308 to 2.177) | 0.4203 | -0.835 (0.919) |
| Model 1 | Asian | 25317 ( 6.4) | 57 | 0.827 (0.767 to 0.888) | 1.903 (1.466 to 2.339) | 0.4636 | 0.240 (0.873) |
| Model 2a | Asian | 25317 ( 6.4) | 57 | 0.800 (0.736 to 0.864) | 1.774 (1.351 to 2.196) | 0.4289 | 0.454 (0.730) |
| Model 2b | Asian | 25317 ( 6.4) | 57 | 0.816 (0.750 to 0.881) | 1.897 (1.471 to 2.323) | 0.4621 | 0.514 (0.780) |
| QRISK@3 | Black | 30763 ( 7.8) | 156 | 0.729 (0.683 to 0.774) | 1.448 (1.176 to 1.721) | 0.3337 | -1.004 (1.023) |
| Model 1 | Black | 30763 ( 7.8) | 156 | 0.742 (0.698 to 0.787) | 1.561 (1.286 to 1.837) | 0.3679 | 0.151 (0.900) |
| Model 2a | Black | 30763 ( 7.8) | 156 | 0.730 (0.688 to 0.772) | 1.384 (1.120 to 1.649) | 0.3139 | 0.946 (0.855) |
| Model 2b | Black | 30763 ( 7.8) | 156 | 0.746 (0.703 to 0.789) | 1.511 (1.244 to 1.778) | 0.3528 | 1.016 (0.899) |
| QRISK@3 | Mixed | 5358 ( 1.4) | 9 | 0.479 (0.266 to 0.692) | 1.490 (0.407 to 2.573) | 0.3464 | -1.189 (0.970) |
| Model 1 | Mixed | 5358 ( 1.4) | 9 | 0.584 (0.445 to 0.724) | 1.726 (0.632 to 2.820) | 0.4156 | -0.047 (0.825) |
| Model 2a | Mixed | 5358 ( 1.4) | 9 | 0.547 (0.351 to 0.743) | 1.621 (0.574 to 2.668) | 0.3855 | 0.176 (0.795) |
| Model 2b | Mixed | 5358 ( 1.4) | 9 | 0.610 (0.446 to 0.774) | 1.800 (0.726 to 2.875) | 0.4362 | 0.247 (0.837) |
| QRISK@3 | Others | 20411 ( 5.2) | 50 | 0.765 (0.698 to 0.832) | 1.565 (1.095 to 2.035) | 0.369 | -1.054 (0.934) |
| Model 1 | Others | 20411 ( 5.2) | 50 | 0.774 (0.709 to 0.839) | 1.729 (1.262 to 2.197) | 0.4166 | 0.049 (0.816) |
| Model 2a | Others | 20411 ( 5.2) | 50 | 0.764 (0.698 to 0.830) | 1.584 (1.123 to 2.045) | 0.3746 | 0.338 (0.776) |
| Model 2b | Others | 20411 ( 5.2) | 50 | 0.774 (0.710 to 0.837) | 1.711 (1.252 to 2.170) | 0.4114 | 0.379 (0.818) |
| QRISK@3 | White | 313777 (79.3) | 775 | 0.716 (0.694 to 0.739) | 1.521 (1.402 to 1.640) | 0.3558 | -1.007 (0.926) |
| Model 1 | White | 313777 (79.3) | 775 | 0.722 (0.700 to 0.745) | 1.589 (1.469 to 1.709) | 0.3762 | 0.116 (0.823) |
| Model 2a | White | 313777 (79.3) | 775 | 0.726 (0.704 to 0.747) | 1.541 (1.425 to 1.657) | 0.3618 | 0.307 (0.784) |
| Model 2b | White | 313777 (79.3) | 775 | 0.729 (0.707 to 0.750) | 1.593 (1.476 to 1.710) | 0.3773 | 0.381 (0.828) |

### Table 9c: Discrimination by Reproductive and pregnancy complications in complete case analysis

| **Model** | **Sub-group** | **Number of women (%)** | **CVD Events within 10 years** | **Harrell's C** | **Royston's D** | **R2D** | **Distribution of LP** |
| --- | --- | --- | --- | --- | --- | --- | --- |
| 3rdQRISK | Endometriosis | 9455 ( 2.4) | 52 | 0.670 (0.584 to 0.757) | 1.182 (0.738 to 1.627) | 0.2502 | -0.651 (0.837) |
| Model 1 | Endometriosis | 9455 ( 2.4) | 52 | 0.681 (0.603 to 0.760) | 1.255 (0.803 to 1.707) | 0.2732 | 0.943 (0.794) |
| Model 2a | Endometriosis | 9455 ( 2.4) | 52 | 0.701 (0.617 to 0.785) | 1.297 (0.857 to 1.738) | 0.2866 | 0.582 (0.775) |
| Model 2b | Endometriosis | 9455 ( 2.4) | 52 | 0.712 (0.636 to 0.789) | 1.358 (0.912 to 1.804) | 0.3057 | 1.215 (0.807) |
| 3rdQRISK | GDM | 12716 ( 3.2) | 65 | 0.765 (0.694 to 0.835) | 1.671 (1.266 to 2.075) | 0.4 | -0.392 (0.947) |
| Model 1 | GDM | 12716 ( 3.2) | 65 | 0.783 (0.718 to 0.848) | 1.736 (1.337 to 2.136) | 0.4185 | 0.833 (0.968) |
| Model 2a | GDM | 12716 ( 3.2) | 65 | 0.765 (0.694 to 0.837) | 1.666 (1.267 to 2.065) | 0.3985 | 0.917 (0.901) |
| Model 2b | GDM | 12716 ( 3.2) | 65 | 0.777 (0.706 to 0.849) | 1.721 (1.321 to 2.122) | 0.4143 | 1.189 (0.938) |
| 3rdQRISK | Gestational hypertension | 15474 ( 3.9) | 83 | 0.777 (0.721 to 0.833) | 1.800 (1.439 to 2.160) | 0.436 | -0.625 (0.963) |
| Model 1 | Gestational hypertension | 15474 ( 3.9) | 83 | 0.786 (0.730 to 0.843) | 1.873 (1.508 to 2.237) | 0.4557 | 0.808 (0.917) |
| Model 2a | Gestational hypertension | 15474 ( 3.9) | 83 | 0.799 (0.752 to 0.846) | 1.823 (1.476 to 2.171) | 0.4425 | 0.716 (0.892) |
| Model 2b | Gestational hypertension | 15474 ( 3.9) | 83 | 0.800 (0.748 to 0.851) | 1.848 (1.503 to 2.194) | 0.4492 | 1.108 (0.911) |
| 3rdQRISK | Irregular menses | 58332 (14.7) | 219 | 0.702 (0.656 to 0.749) | 1.434 (1.211 to 1.657) | 0.3292 | -0.897 (0.963) |
| Model 1 | Irregular menses | 58332 (14.7) | 219 | 0.716 (0.671 to 0.761) | 1.527 (1.302 to 1.752) | 0.3577 | 0.440 (0.885) |
| Model 2a | Irregular menses | 58332 (14.7) | 219 | 0.712 (0.667 to 0.757) | 1.487 (1.269 to 1.706) | 0.3456 | 0.497 (0.854) |
| Model 2b | Irregular menses | 58332 (14.7) | 219 | 0.723 (0.679 to 0.767) | 1.551 (1.329 to 1.773) | 0.3648 | 0.631 (0.907) |
| 3rdQRISK | Miscarriage | 91657 (23.2) | 357 | 0.707 (0.675 to 0.740) | 1.402 (1.227 to 1.576) | 0.3193 | -0.680 (0.918) |
| Model 1 | Miscarriage | 91657 (23.2) | 357 | 0.725 (0.693 to 0.756) | 1.526 (1.350 to 1.702) | 0.3573 | 0.497 (0.865) |
| Model 2a | Miscarriage | 91657 (23.2) | 357 | 0.728 (0.697 to 0.759) | 1.490 (1.320 to 1.661) | 0.3466 | 0.609 (0.820) |
| Model 2b | Miscarriage | 91657 (23.2) | 357 | 0.739 (0.708 to 0.769) | 1.578 (1.406 to 1.750) | 0.3729 | 0.812 (0.858) |
| 3rdQRISK | PCOS | 16641 ( 4.2) | 60 | 0.689 (0.598 to 0.780) | 1.204 (0.777 to 1.631) | 0.2571 | -0.789 (0.889) |
| Model 1 | PCOS | 16641 ( 4.2) | 60 | 0.697 (0.611 to 0.784) | 1.221 (0.793 to 1.649) | 0.2624 | 0.634 (0.851) |
| Model 2a | PCOS | 16641 ( 4.2) | 60 | 0.700 (0.619 to 0.780) | 1.331 (0.915 to 1.746) | 0.2971 | 0.559 (0.817) |
| Model 2b | PCOS | 16641 ( 4.2) | 60 | 0.709 (0.630 to 0.787) | 1.380 (0.961 to 1.799) | 0.3125 | 0.939 (0.862) |
| 3rdQRISK | Placental abruption | 2193 ( 0.6) | 9 | 0.827 (0.686 to 0.968) | 1.472 (0.442 to 2.502) | 0.3409 | -0.782 (0.913) |
| Model 1 | Placental abruption | 2193 ( 0.6) | 9 | 0.797 (0.658 to 0.937) | 1.419 (0.379 to 2.459) | 0.3246 | 0.614 (0.894) |
| Model 2a | Placental abruption | 2193 ( 0.6) | 9 | 0.787 (0.639 to 0.934) | 1.320 (0.308 to 2.333) | 0.2939 | 0.570 (0.802) |
| Model 2b | Placental abruption | 2193 ( 0.6) | 9 | 0.759 (0.613 to 0.905) | 1.137 (0.111 to 2.163) | 0.2359 | 0.760 (0.875) |
| 3rdQRISK | Postpartum depression | 23331 ( 5.9) | 96 | 0.752 (0.700 to 0.805) | 1.776 (1.439 to 2.112) | 0.4295 | -0.908 (0.934) |
| Model 1 | Postpartum depression | 23331 ( 5.9) | 96 | 0.755 (0.704 to 0.807) | 1.802 (1.465 to 2.140) | 0.4367 | 0.546 (0.867) |
| Model 2a | Postpartum depression | 23331 ( 5.9) | 96 | 0.764 (0.709 to 0.818) | 1.795 (1.468 to 2.123) | 0.4349 | 0.508 (0.823) |
| Model 2b | Postpartum depression | 23331 ( 5.9) | 96 | 0.767 (0.715 to 0.819) | 1.831 (1.499 to 2.163) | 0.4446 | 0.879 (0.876) |
| 3rdQRISK | Preeclampsia | 14150 ( 3.6) | 75 | 0.796 (0.732 to 0.860) | 1.912 (1.537 to 2.288) | 0.4661 | -0.714 (0.942) |
| Model 1 | Preeclampsia | 14150 ( 3.6) | 75 | 0.802 (0.739 to 0.864) | 2.000 (1.625 to 2.376) | 0.4885 | 0.780 (0.884) |
| Model 2a | Preeclampsia | 14150 ( 3.6) | 75 | 0.813 (0.757 to 0.870) | 1.947 (1.574 to 2.320) | 0.4751 | 0.640 (0.853) |
| Model 2b | Preeclampsia | 14150 ( 3.6) | 75 | 0.819 (0.763 to 0.875) | 2.037 (1.660 to 2.413) | 0.4975 | 1.119 (0.873) |
| 3rdQRISK | Preterm birth | 20029 ( 5.1) | 88 | 0.793 (0.735 to 0.851) | 2.050 (1.697 to 2.403) | 0.5008 | -0.849 (0.945) |
| Model 1 | Preterm birth | 20029 ( 5.1) | 88 | 0.799 (0.744 to 0.855) | 2.047 (1.700 to 2.394) | 0.5001 | 0.627 (0.899) |
| Model 2a | Preterm birth | 20029 ( 5.1) | 88 | 0.793 (0.732 to 0.855) | 2.038 (1.695 to 2.382) | 0.498 | 0.502 (0.839) |
| Model 2b | Preterm birth | 20029 ( 5.1) | 88 | 0.798 (0.739 to 0.858) | 2.059 (1.711 to 2.407) | 0.5031 | 0.668 (0.911) |
| 3rdQRISK | SGA | 26053 ( 6.6) | 55 | 0.692 (0.596 to 0.789) | 1.359 (0.911 to 1.807) | 0.306 | -1.074 (0.901) |
| Model 1 | SGA | 26053 ( 6.6) | 55 | 0.712 (0.622 to 0.801) | 1.508 (1.060 to 1.957) | 0.352 | 0.087 (0.813) |
| Model 2a | SGA | 26053 ( 6.6) | 55 | 0.674 (0.576 to 0.771) | 1.354 (0.915 to 1.793) | 0.3044 | 0.319 (0.768) |
| Model 2b | SGA | 26053 ( 6.6) | 55 | 0.690 (0.598 to 0.783) | 1.490 (1.047 to 1.933) | 0.3465 | 0.421 (0.827) |
| 3rdQRISK | Stillbirth | 3863 ( 1.0) | 21 | 0.780 (0.654 to 0.906) | 1.921 (1.209 to 2.633) | 0.4684 | -0.624 (0.945) |
| Model 1 | Stillbirth | 3863 ( 1.0) | 21 | 0.773 (0.635 to 0.911) | 1.789 (1.102 to 2.476) | 0.4331 | 0.740 (0.938) |
| Model 2a | Stillbirth | 3863 ( 1.0) | 21 | 0.787 (0.668 to 0.906) | 1.868 (1.186 to 2.551) | 0.4546 | 0.725 (0.863) |
| Model 2b | Stillbirth | 3863 ( 1.0) | 21 | 0.784 (0.666 to 0.902) | 1.812 (1.125 to 2.499) | 0.4393 | 1.057 (0.923) |

### Table 10: Overall discrimination in sensitivity analysis restricting to those aged 25 years of age and above

| Model | Harrell's C | Royston's D | R2D | Distribution of LP |
| --- | --- | --- | --- | --- |
| QRISK3 | 0.688 (0.672 to 0.704) | 1.260 (1.177 to 1.343) | 0.2748 | -0.869 (0.822) |
| Model 1 | 0.702 (0.686 to 0.718) | 1.380 (1.297 to 1.465) | 0.3129 | 0.113 (0.697) |
| Model 2a | 0.706 (0.690 to 0.721) | 1.340 (1.259 to 1.421) | 0.3 | 0.230 (0.676) |
| Model 2b | 0.715 (0.700 to 0.731) | 1.437 (1.356 to 1.520) | 0.3303 | 0.323 (0.729) |

### Table 11a: Discrimination by Age groups in sensitivity analysis restricting to those aged 25 years of age and above

| Model | Sub-group | Number of women (%) | CVD Events within 10 years | Harrell's C | Royston's D | R2D | Distribution of LP |
| --- | --- | --- | --- | --- | --- | --- | --- |
| 3rdQRISK | 25-34 | 272418 (57.9) | 526 | 0.597 (0.567 to 0.628) | 0.697 (0.556 to 0.838) | 0.1039 | -1.339 (0.644) |
| Model 1 | 25-34 | 272418 (57.9) | 526 | 0.630 (0.600 to 0.660) | 0.900 (0.758 to 1.042) | 0.1621 | -0.249 (0.521) |
| Model 2a | 25-34 | 272418 (57.9) | 526 | 0.620 (0.590 to 0.649) | 0.833 (0.697 to 0.970) | 0.1422 | -0.039 (0.564) |
| Model 2b | 25-34 | 272418 (57.9) | 526 | 0.642 (0.613 to 0.671) | 0.981 (0.842 to 1.119) | 0.1867 | 0.041 (0.618) |
| 3rdQRISK | 35-49 | 198128 (42.1) | 1014 | 0.706 (0.688 to 0.724) | 1.318 (1.219 to 1.418) | 0.2933 | -0.227 (0.570) |
| Model 1 | 35-49 | 198128 (42.1) | 1014 | 0.714 (0.695 to 0.732) | 1.398 (1.297 to 1.499) | 0.3181 | 0.617 (0.613) |
| Model 2a | 35-49 | 198128 (42.1) | 1014 | 0.724 (0.708 to 0.741) | 1.405 (1.306 to 1.504) | 0.3203 | 0.592 (0.647) |
| Model 2b | 35-49 | 198128 (42.1) | 1014 | 0.729 (0.712 to 0.747) | 1.479 (1.378 to 1.579) | 0.3429 | 0.702 (0.697) |

### Table 11b: Discrimination by Ethnicity in sensitivity analysis restricting to those aged 25 years of age and above

| Model | Sub-group | Number of women (%) | CVD Events within 10 years | Harrell's C | Royston's D | R2D | Distribution of LP |
| --- | --- | --- | --- | --- | --- | --- | --- |
| 3rdQRISK | Asian | 32070 ( 6.8) | 90 | 0.816 (0.764 to 0.868) | 1.908 (1.565 to 2.251) | 0.4651 | -0.790 (0.834) |
| Model 1 | Asian | 32070 ( 6.8) | 90 | 0.839 (0.792 to 0.885) | 2.046 (1.700 to 2.392) | 0.4998 | 0.157 (0.754) |
| Model 2a | Asian | 32070 ( 6.8) | 90 | 0.820 (0.772 to 0.868) | 1.841 (1.508 to 2.174) | 0.4472 | 0.281 (0.622) |
| Model 2b | Asian | 32070 ( 6.8) | 90 | 0.838 (0.792 to 0.883) | 1.976 (1.641 to 2.312) | 0.4826 | 0.359 (0.687) |
| 3rdQRISK | Black | 36805 ( 7.8) | 232 | 0.705 (0.668 to 0.743) | 1.242 (1.021 to 1.462) | 0.2691 | -0.903 (0.904) |
| Model 1 | Black | 36805 ( 7.8) | 232 | 0.719 (0.681 to 0.756) | 1.346 (1.124 to 1.568) | 0.302 | 0.118 (0.767) |
| Model 2a | Black | 36805 ( 7.8) | 232 | 0.692 (0.654 to 0.730) | 1.119 (0.908 to 1.331) | 0.2303 | 0.836 (0.710) |
| Model 2b | Black | 36805 ( 7.8) | 232 | 0.706 (0.667 to 0.744) | 1.228 (1.013 to 1.442) | 0.2646 | 0.927 (0.763) |
| 3rdQRISK | Mixed | 6033 ( 1.3) | 20 | 0.536 (0.370 to 0.701) | 1.167 (0.446 to 1.888) | 0.2453 | -0.987 (0.848) |
| Model 1 | Mixed | 6033 ( 1.3) | 20 | 0.618 (0.468 to 0.767) | 1.386 (0.660 to 2.112) | 0.3144 | 0.012 (0.702) |
| Model 2a | Mixed | 6033 ( 1.3) | 20 | 0.581 (0.431 to 0.731) | 1.367 (0.665 to 2.069) | 0.3085 | 0.471 (0.654) |
| Model 2b | Mixed | 6033 ( 1.3) | 20 | 0.648 (0.498 to 0.798) | 1.576 (0.860 to 2.293) | 0.3724 | 0.565 (0.706) |
| 3rdQRISK | Others | 25881 ( 5.5) | 71 | 0.724 (0.652 to 0.795) | 1.343 (0.953 to 1.734) | 0.3011 | -1.009 (0.844) |
| Model 1 | Others | 25881 ( 5.5) | 71 | 0.733 (0.663 to 0.804) | 1.473 (1.083 to 1.862) | 0.3411 | -0.017 (0.697) |
| Model 2a | Others | 25881 ( 5.5) | 71 | 0.739 (0.672 to 0.806) | 1.434 (1.055 to 1.814) | 0.3294 | 0.106 (0.650) |
| Model 2b | Others | 25881 ( 5.5) | 71 | 0.752 (0.686 to 0.817) | 1.540 (1.161 to 1.920) | 0.3616 | 0.177 (0.702) |
| 3rdQRISK | Unknown | 14996 ( 3.2) | 12 | 0.756 (0.661 to 0.851) | 1.659 (0.739 to 2.578) | 0.3964 | -0.957 (0.827) |
| Model 1 | Unknown | 14996 ( 3.2) | 12 | 0.744 (0.635 to 0.853) | 1.754 (0.825 to 2.684) | 0.4235 | -0.055 (0.652) |
| Model 2a | Unknown | 14996 ( 3.2) | 12 | 0.761 (0.666 to 0.856) | 1.560 (0.662 to 2.458) | 0.3676 | 0.086 (0.619) |
| Model 2b | Unknown | 14996 ( 3.2) | 12 | 0.748 (0.642 to 0.855) | 1.638 (0.727 to 2.549) | 0.3905 | 0.081 (0.636) |
| 3rdQRISK | White | 354761 (75.4) | 1115 | 0.674 (0.654 to 0.693) | 1.197 (1.099 to 1.294) | 0.2548 | -0.859 (0.810) |
| Model 1 | White | 354761 (75.4) | 1115 | 0.686 (0.667 to 0.705) | 1.302 (1.203 to 1.400) | 0.288 | 0.130 (0.695) |
| Model 2a | White | 354761 (75.4) | 1115 | 0.689 (0.670 to 0.707) | 1.259 (1.164 to 1.354) | 0.2745 | 0.169 (0.649) |
| Model 2b | White | 354761 (75.4) | 1115 | 0.697 (0.678 to 0.715) | 1.347 (1.250 to 1.443) | 0.3021 | 0.269 (0.704) |

### Table 11c: Discrimination by reproductive factors and pregnancy complications

| Model | Sub-group | Number of women (%) | CVD Events within 10 years | Harrell's C | Royston's D | R2D | Distribution of LP |
| --- | --- | --- | --- | --- | --- | --- | --- |
| 3rdQRISK | Endometriosis | 11893 ( 2.5) | 67 | 0.646 (0.571 to 0.721) | 1.001 (0.609 to 1.394) | 0.1931 | -0.643 (0.777) |
| Model 1 | Endometriosis | 11893 ( 2.5) | 67 | 0.680 (0.613 to 0.747) | 1.161 (0.764 to 1.558) | 0.2435 | 0.717 (0.696) |
| Model 2a | Endometriosis | 11893 ( 2.5) | 67 | 0.682 (0.608 to 0.756) | 1.189 (0.803 to 1.575) | 0.2523 | 0.331 (0.682) |
| Model 2b | Endometriosis | 11893 ( 2.5) | 67 | 0.704 (0.635 to 0.773) | 1.311 (0.917 to 1.704) | 0.2908 | 0.891 (0.730) |
| 3rdQRISK | GDM | 15571 ( 3.3) | 92 | 0.733 (0.671 to 0.795) | 1.450 (1.115 to 1.785) | 0.3343 | -0.413 (0.906) |
| Model 1 | GDM | 15571 ( 3.3) | 92 | 0.755 (0.696 to 0.815) | 1.553 (1.219 to 1.888) | 0.3655 | 0.760 (0.866) |
| Model 2a | GDM | 15571 ( 3.3) | 92 | 0.735 (0.671 to 0.798) | 1.411 (1.082 to 1.739) | 0.3221 | 0.642 (0.836) |
| Model 2b | GDM | 15571 ( 3.3) | 92 | 0.753 (0.688 to 0.818) | 1.522 (1.191 to 1.854) | 0.3562 | 0.963 (0.883) |
| 3rdQRISK | Gestational hypertension | 18082 ( 3.8) | 119 | 0.747 (0.693 to 0.802) | 1.607 (1.308 to 1.905) | 0.3813 | -0.571 (0.878) |
| Model 1 | Gestational hypertension | 18082 ( 3.8) | 119 | 0.762 (0.711 to 0.813) | 1.677 (1.381 to 1.974) | 0.4018 | 0.805 (0.791) |
| Model 2a | Gestational hypertension | 18082 ( 3.8) | 119 | 0.770 (0.722 to 0.818) | 1.654 (1.368 to 1.940) | 0.395 | 0.493 (0.792) |
| Model 2b | Gestational hypertension | 18082 ( 3.8) | 119 | 0.778 (0.731 to 0.825) | 1.727 (1.441 to 2.013) | 0.416 | 0.987 (0.817) |
| 3rdQRISK | Irregular menses | 58012 (12.3) | 237 | 0.703 (0.660 to 0.746) | 1.379 (1.166 to 1.591) | 0.3122 | -0.745 (0.868) |
| Model 1 | Irregular menses | 58012 (12.3) | 237 | 0.721 (0.680 to 0.762) | 1.489 (1.274 to 1.704) | 0.3461 | 0.347 (0.778) |
| Model 2a | Irregular menses | 58012 (12.3) | 237 | 0.717 (0.676 to 0.757) | 1.458 (1.250 to 1.666) | 0.3368 | 0.365 (0.748) |
| Model 2b | Irregular menses | 58012 (12.3) | 237 | 0.730 (0.691 to 0.769) | 1.528 (1.316 to 1.740) | 0.358 | 0.541 (0.810) |
| 3rdQRISK | Miscarriage | 114873 (24.4) | 545 | 0.687 (0.661 to 0.714) | 1.225 (1.086 to 1.365) | 0.2639 | -0.628 (0.832) |
| Model 1 | Miscarriage | 114873 (24.4) | 545 | 0.703 (0.678 to 0.729) | 1.345 (1.204 to 1.485) | 0.3015 | 0.472 (0.735) |
| Model 2a | Miscarriage | 114873 (24.4) | 545 | 0.711 (0.686 to 0.736) | 1.349 (1.213 to 1.486) | 0.303 | 0.388 (0.711) |
| Model 2b | Miscarriage | 114873 (24.4) | 545 | 0.721 (0.695 to 0.746) | 1.448 (1.310 to 1.586) | 0.3335 | 0.670 (0.753) |
| 3rdQRISK | PCOS | 19530 ( 4.2) | 86 | 0.702 (0.629 to 0.775) | 1.251 (0.898 to 1.604) | 0.2719 | -0.749 (0.823) |
| Model 1 | PCOS | 19530 ( 4.2) | 86 | 0.710 (0.641 to 0.779) | 1.287 (0.932 to 1.642) | 0.2834 | 0.597 (0.746) |
| Model 2a | PCOS | 19530 ( 4.2) | 86 | 0.732 (0.672 to 0.793) | 1.445 (1.101 to 1.789) | 0.3325 | 0.331 (0.728) |
| Model 2b | PCOS | 19530 ( 4.2) | 86 | 0.736 (0.676 to 0.796) | 1.482 (1.134 to 1.830) | 0.3441 | 0.785 (0.781) |
| 3rdQRISK | Placental abruption | 2640 ( 0.6) | 14 | 0.663 (0.478 to 0.848) | 0.640 (-0.197 to 1.478) | 0.0891 | -0.724 (0.824) |
| Model 1 | Placental abruption | 2640 ( 0.6) | 14 | 0.648 (0.467 to 0.830) | 0.682 (-0.159 to 1.523) | 0.1 | 0.648 (0.785) |
| Model 2a | Placental abruption | 2640 ( 0.6) | 14 | 0.684 (0.508 to 0.859) | 0.870 (0.059 to 1.682) | 0.1532 | 0.368 (0.702) |
| Model 2b | Placental abruption | 2640 ( 0.6) | 14 | 0.671 (0.500 to 0.842) | 0.800 (-0.022 to 1.622) | 0.1326 | 0.611 (0.803) |
| 3rdQRISK | Postpartum depression | 26055 ( 5.5) | 135 | 0.702 (0.651 to 0.753) | 1.382 (1.103 to 1.662) | 0.3132 | -0.794 (0.831) |
| Model 1 | Postpartum depression | 26055 ( 5.5) | 135 | 0.700 (0.650 to 0.751) | 1.395 (1.114 to 1.676) | 0.3172 | 0.575 (0.740) |
| Model 2a | Postpartum depression | 26055 ( 5.5) | 135 | 0.721 (0.670 to 0.772) | 1.454 (1.183 to 1.725) | 0.3354 | 0.321 (0.709) |
| Model 2b | Postpartum depression | 26055 ( 5.5) | 135 | 0.722 (0.672 to 0.771) | 1.488 (1.213 to 1.763) | 0.3459 | 0.774 (0.773) |
| 3rdQRISK | Preeclampsia | 16611 ( 3.5) | 105 | 0.754 (0.700 to 0.809) | 1.605 (1.292 to 1.919) | 0.3809 | -0.648 (0.857) |
| Model 1 | Preeclampsia | 16611 ( 3.5) | 105 | 0.762 (0.709 to 0.815) | 1.688 (1.374 to 2.003) | 0.405 | 0.756 (0.760) |
| Model 2a | Preeclampsia | 16611 ( 3.5) | 105 | 0.771 (0.721 to 0.820) | 1.615 (1.308 to 1.921) | 0.3837 | 0.434 (0.754) |
| Model 2b | Preeclampsia | 16611 ( 3.5) | 105 | 0.776 (0.726 to 0.827) | 1.704 (1.393 to 2.015) | 0.4094 | 0.962 (0.781) |
| 3rdQRISK | Preterm birth | 20540 ( 4.4) | 96 | 0.758 (0.694 to 0.821) | 1.812 (1.481 to 2.143) | 0.4395 | -0.724 (0.852) |
| Model 1 | Preterm birth | 20540 ( 4.4) | 96 | 0.761 (0.698 to 0.823) | 1.806 (1.474 to 2.139) | 0.4378 | 0.510 (0.798) |
| Model 2a | Preterm birth | 20540 ( 4.4) | 96 | 0.763 (0.699 to 0.828) | 1.839 (1.515 to 2.162) | 0.4466 | 0.360 (0.743) |
| Model 2b | Preterm birth | 20540 ( 4.4) | 96 | 0.764 (0.700 to 0.828) | 1.845 (1.516 to 2.175) | 0.4484 | 0.572 (0.833) |
| 3rdQRISK | SGA | 28634 ( 6.1) | 73 | 0.638 (0.552 to 0.723) | 1.102 (0.721 to 1.483) | 0.2247 | -0.933 (0.796) |
| Model 1 | SGA | 28634 ( 6.1) | 73 | 0.664 (0.582 to 0.746) | 1.296 (0.914 to 1.679) | 0.2863 | 0.050 (0.700) |
| Model 2a | SGA | 28634 ( 6.1) | 73 | 0.632 (0.547 to 0.716) | 1.175 (0.802 to 1.548) | 0.248 | 0.193 (0.654) |
| Model 2b | SGA | 28634 ( 6.1) | 73 | 0.658 (0.574 to 0.743) | 1.360 (0.983 to 1.737) | 0.3063 | 0.328 (0.729) |
| 3rdQRISK | Stillbirth | 4788 ( 1.0) | 37 | 0.677 (0.559 to 0.795) | 1.093 (0.555 to 1.630) | 0.2219 | -0.586 (0.872) |
| Model 1 | Stillbirth | 4788 ( 1.0) | 37 | 0.693 (0.583 to 0.804) | 1.184 (0.649 to 1.719) | 0.2507 | 0.887 (0.821) |
| Model 2a | Stillbirth | 4788 ( 1.0) | 37 | 0.702 (0.592 to 0.812) | 1.121 (0.601 to 1.641) | 0.2307 | 0.497 (0.770) |
| Model 2b | Stillbirth | 4788 ( 1.0) | 37 | 0.716 (0.612 to 0.821) | 1.213 (0.685 to 1.740) | 0.2599 | 1.077 (0.840) |

## Table 12: Overall discrimination in sensitivity analysis using multiple imputation

| Model | Harrell's C | Royston's D |
| --- | --- | --- |
| QRISK3 | 0.705 (0.689 to 0.721) | 1.362 (1.277 to 1.446) |
| Model 1 | 0.717 (0.702 to 0.732) | 1.464 (1.379 to 1.549) |
| Model 2a | 0.718 (0.703 to 0.734) | 1.416 (1.331 to 1.500) |
| Model 2b | 0.727 (0.712 to 0.742) | 1.504 (1.418 to 1.590) |

## Calibration plots by subgroup in main analysis

### Fig. S1: Calibration plots by Age groups in main analysis

#### Fig. S1a: Age 15 to 34

**QRISK3** – original QRISK3 model, **QRISK3-R** – Recalibrated QRISK3 model, **Model 1** – Updated model with QRISK3 predictors only, **Model 2a** – Updated model with QRISK3 linear predictor plus additional reproductive and pregnancy complications, **Model 2b** – Updated model with all predictors (QRISK3 predictors plus reproductive and pregnancy complications.

#### Fig. S1b: Age 35 to 49

**QRISK3** – original QRISK3 model, **QRISK3-R** – Recalibrated QRISK3 model, **Model 1** – Updated model with QRISK3 predictors only, **Model 2a** – Updated model with QRISK3 linear predictor plus additional reproductive and pregnancy complications, **Model 2b** – Updated model with all predictors (QRISK3 predictors plus reproductive and pregnancy complications.

### Fig. S2: Calibration plots by Ethnicity in main analysis

#### Fig. S2a: White ethnicity

**QRISK3** – original QRISK3 model, **QRISK3-R** – Recalibrated QRISK3 model, **Model 1** – Updated model with QRISK3 predictors only, **Model 2a** – Updated model with QRISK3 linear predictor plus additional reproductive and pregnancy complications, **Model 2b** – Updated model with all predictors (QRISK3 predictors plus reproductive and pregnancy complications.

#### Fig. S2b: Black ethnicity

**QRISK3** – original QRISK3 model, **QRISK3-R** – Recalibrated QRISK3 model, **Model 1** – Updated model with QRISK3 predictors only, **Model 2a** – Updated model with QRISK3 linear predictor plus additional reproductive and pregnancy complications, **Model 2b** – Updated model with all predictors (QRISK3 predictors plus reproductive and pregnancy complications.

#### Fig. S2c: Asian ethnicity

**QRISK3** – original QRISK3 model, **QRISK3-R** – Recalibrated QRISK3 model, **Model 1** – Updated model with QRISK3 predictors only, **Model 2a** – Updated model with QRISK3 linear predictor plus additional reproductive and pregnancy complications, **Model 2b** – Updated model with all predictors (QRISK3 predictors plus reproductive and pregnancy complications.

#### Fig. S2d: Mixed ethnicity

**QRISK3** – original QRISK3 model, **QRISK3-R** – Recalibrated QRISK3 model, **Model 1** – Updated model with QRISK3 predictors only, **Model 2a** – Updated model with QRISK3 linear predictor plus additional reproductive and pregnancy complications, **Model 2b** – Updated model with all predictors (QRISK3 predictors plus reproductive and pregnancy complications.

#### Fig. S2e: Other ethnicity

**QRISK3** – original QRISK3 model, **QRISK3-R** – Recalibrated QRISK3 model, **Model 1** – Updated model with QRISK3 predictors only, **Model 2a** – Updated model with QRISK3 linear predictor plus additional reproductive and pregnancy complications, **Model 2b** – Updated model with all predictors (QRISK3 predictors plus reproductive and pregnancy complications.

#### Fig. S2f: Unknown ethnicity

**QRISK3** – original QRISK3 model, **QRISK3-R** – Recalibrated QRISK3 model, **Model 1** – Updated model with QRISK3 predictors only, **Model 2a** – Updated model with QRISK3 linear predictor plus additional reproductive and pregnancy complications, **Model 2b** – Updated model with all predictors (QRISK3 predictors plus reproductive and pregnancy complications.

### Fig. S3: Calibration plots by Reproductive and pregnancy complications in main analysis

#### Fig. S3a: PCOS

**QRISK3** – original QRISK3 model, **QRISK3-R** – Recalibrated QRISK3 model, **Model 1** – Updated model with QRISK3 predictors only, **Model 2a** – Updated model with QRISK3 linear predictor plus additional reproductive and pregnancy complications, **Model 2b** – Updated model with all predictors (QRISK3 predictors plus reproductive and pregnancy complications.

#### Fig. S3b: Irregular menses

**QRISK3** – original QRISK3 model, **QRISK3-R** – Recalibrated QRISK3 model, **Model 1** – Updated model with QRISK3 predictors only, **Model 2a** – Updated model with QRISK3 linear predictor plus additional reproductive and pregnancy complications, **Model 2b** – Updated model with all predictors (QRISK3 predictors plus reproductive and pregnancy complications.

#### Fig. S3c: Endometriosis

**QRISK3** – original QRISK3 model, **QRISK3-R** – Recalibrated QRISK3 model, **Model 1** – Updated model with QRISK3 predictors only, **Model 2a** – Updated model with QRISK3 linear predictor plus additional reproductive and pregnancy complications, **Model 2b** – Updated model with all predictors (QRISK3 predictors plus reproductive and pregnancy complications.

#### Fig. S3d: Miscarriage

**QRISK3** – original QRISK3 model, **QRISK3-R** – Recalibrated QRISK3 model, **Model 1** – Updated model with QRISK3 predictors only, **Model 2a** – Updated model with QRISK3 linear predictor plus additional reproductive and pregnancy complications, **Model 2b** – Updated model with all predictors (QRISK3 predictors plus reproductive and pregnancy complications.

#### Fig. S3e: Gestational hypertension

**QRISK3** – original QRISK3 model, **QRISK3-R** – Recalibrated QRISK3 model, **Model 1** – Updated model with QRISK3 predictors only, **Model 2a** – Updated model with QRISK3 linear predictor plus additional reproductive and pregnancy complications, **Model 2b** – Updated model with all predictors (QRISK3 predictors plus reproductive and pregnancy complications.

#### Fig. S3f: Pre-eclampsia

**QRISK3** – original QRISK3 model, **QRISK3-R** – Recalibrated QRISK3 model, **Model 1** – Updated model with QRISK3 predictors only, **Model 2a** – Updated model with QRISK3 linear predictor plus additional reproductive and pregnancy complications, **Model 2b** – Updated model with all predictors (QRISK3 predictors plus reproductive and pregnancy complications.

#### Fig. S3g: GDM

**QRISK3** – original QRISK3 model, **QRISK3-R** – Recalibrated QRISK3 model, **Model 1** – Updated model with QRISK3 predictors only, **Model 2a** – Updated model with QRISK3 linear predictor plus additional reproductive and pregnancy complications, **Model 2b** – Updated model with all predictors (QRISK3 predictors plus reproductive and pregnancy complications.

#### Fig. S3h: Preterm birth

**QRISK3** – original QRISK3 model, **QRISK3-R** – Recalibrated QRISK3 model, **Model 1** – Updated model with QRISK3 predictors only, **Model 2a** – Updated model with QRISK3 linear predictor plus additional reproductive and pregnancy complications, **Model 2b** – Updated model with all predictors (QRISK3 predictors plus reproductive and pregnancy complications.

#### Fig. S3i: Stillbirth

**QRISK3** – original QRISK3 model, **QRISK3-R** – Recalibrated QRISK3 model, **Model 1** – Updated model with QRISK3 predictors only, **Model 2a** – Updated model with QRISK3 linear predictor plus additional reproductive and pregnancy complications, **Model 2b** – Updated model with all predictors (QRISK3 predictors plus reproductive and pregnancy complications.

#### Fig. S3j: Placental abruption

**QRISK3** – original QRISK3 model, **QRISK3-R** – Recalibrated QRISK3 model, **Model 1** – Updated model with QRISK3 predictors only, **Model 2a** – Updated model with QRISK3 linear predictor plus additional reproductive and pregnancy complications, **Model 2b** – Updated model with all predictors (QRISK3 predictors plus reproductive and pregnancy complications.

#### Fig. S3k: SGA

**QRISK3** – original QRISK3 model, **QRISK3-R** – Recalibrated QRISK3 model, **Model 1** – Updated model with QRISK3 predictors only, **Model 2a** – Updated model with QRISK3 linear predictor plus additional reproductive and pregnancy complications, **Model 2b** – Updated model with all predictors (QRISK3 predictors plus reproductive and pregnancy complications.

#### Fig. S3l: Postnatal depression

**QRISK3** – original QRISK3 model, **QRISK3-R** – Recalibrated QRISK3 model, **Model 1** – Updated model with QRISK3 predictors only, **Model 2a** – Updated model with QRISK3 linear predictor plus additional reproductive and pregnancy complications, **Model 2b** – Updated model with all predictors (QRISK3 predictors plus reproductive and pregnancy complications.

## Calibration plots overall and by subgroup in complete case analysis

### Fig. S4: Overall Calibration plot

**QRISK3** – original QRISK3 model, **QRISK3-R** – Recalibrated QRISK3 model, **Model 1** – Updated model with QRISK3 predictors only, **Model 2a** – Updated model with QRISK3 linear predictor plus additional reproductive and pregnancy complications, **Model 2b** – Updated model with all predictors (QRISK3 predictors plus reproductive and pregnancy complications.

### Fig. S5: Age groups

#### Fig. S5a: Age 15 - 34

**QRISK3** – original QRISK3 model, **QRISK3-R** – Recalibrated QRISK3 model, **Model 1** – Updated model with QRISK3 predictors only, **Model 2a** – Updated model with QRISK3 linear predictor plus additional reproductive and pregnancy complications, **Model 2b** – Updated model with all predictors (QRISK3 predictors plus reproductive and pregnancy complications.

#### Fig. S5b: Age 35 – 49

**QRISK3** – original QRISK3 model, **QRISK3-R** – Recalibrated QRISK3 model, **Model 1** – Updated model with QRISK3 predictors only, **Model 2a** – Updated model with QRISK3 linear predictor plus additional reproductive and pregnancy complications, **Model 2b** – Updated model with all predictors (QRISK3 predictors plus reproductive and pregnancy complications.

### Fig. S6: Ethnicity

#### Fig. S6a: White

**QRISK3** – original QRISK3 model, **QRISK3-R** – Recalibrated QRISK3 model, **Model 1** – Updated model with QRISK3 predictors only, **Model 2a** – Updated model with QRISK3 linear predictor plus additional reproductive and pregnancy complications, **Model 2b** – Updated model with all predictors (QRISK3 predictors plus reproductive and pregnancy complications.

#### Fig. S6b: Black ethnicity

**QRISK3** – original QRISK3 model, **QRISK3-R** – Recalibrated QRISK3 model, **Model 1** – Updated model with QRISK3 predictors only, **Model 2a** – Updated model with QRISK3 linear predictor plus additional reproductive and pregnancy complications, **Model 2b** – Updated model with all predictors (QRISK3 predictors plus reproductive and pregnancy complications.

#### Fig. S6c: Asian ethnicity

**QRISK3** – original QRISK3 model, **QRISK3-R** – Recalibrated QRISK3 model, **Model 1** – Updated model with QRISK3 predictors only, **Model 2a** – Updated model with QRISK3 linear predictor plus additional reproductive and pregnancy complications, **Model 2b** – Updated model with all predictors (QRISK3 predictors plus reproductive and pregnancy complications.

#### Fig. S6d: Mixed ethnicity

**QRISK3** – original QRISK3 model, **QRISK3-R** – Recalibrated QRISK3 model, **Model 1** – Updated model with QRISK3 predictors only, **Model 2a** – Updated model with QRISK3 linear predictor plus additional reproductive and pregnancy complications, **Model 2b** – Updated model with all predictors (QRISK3 predictors plus reproductive and pregnancy complications.

#### Fig. S6e: Other ethnicity

**QRISK3** – original QRISK3 model, **QRISK3-R** – Recalibrated QRISK3 model, **Model 1** – Updated model with QRISK3 predictors only, **Model 2a** – Updated model with QRISK3 linear predictor plus additional reproductive and pregnancy complications, **Model 2b** – Updated model with all predictors (QRISK3 predictors plus reproductive and pregnancy complications.

### Fig. S7: Reproductive and pregnancy complications

#### Fig. S7a: PCOS

**QRISK3** – original QRISK3 model, **QRISK3-R** – Recalibrated QRISK3 model, **Model 1** – Updated model with QRISK3 predictors only, **Model 2a** – Updated model with QRISK3 linear predictor plus additional reproductive and pregnancy complications, **Model 2b** – Updated model with all predictors (QRISK3 predictors plus reproductive and pregnancy complications.

#### Fig. S7b: Irregular menses

**QRISK3** – original QRISK3 model, **QRISK3-R** – Recalibrated QRISK3 model, **Model 1** – Updated model with QRISK3 predictors only, **Model 2a** – Updated model with QRISK3 linear predictor plus additional reproductive and pregnancy complications, **Model 2b** – Updated model with all predictors (QRISK3 predictors plus reproductive and pregnancy complications.

#### Fig. S7c: Endometriosis

**QRISK3** – original QRISK3 model, **QRISK3-R** – Recalibrated QRISK3 model, **Model 1** – Updated model with QRISK3 predictors only, **Model 2a** – Updated model with QRISK3 linear predictor plus additional reproductive and pregnancy complications, **Model 2b** – Updated model with all predictors (QRISK3 predictors plus reproductive and pregnancy complications.

#### Fig. S7d: Miscarriage

**QRISK3** – original QRISK3 model, **QRISK3-R** – Recalibrated QRISK3 model, **Model 1** – Updated model with QRISK3 predictors only, **Model 2a** – Updated model with QRISK3 linear predictor plus additional reproductive and pregnancy complications, **Model 2b** – Updated model with all predictors (QRISK3 predictors plus reproductive and pregnancy complications.

#### Fig. S7e: Gestational hypertension

**QRISK3** – original QRISK3 model, **QRISK3-R** – Recalibrated QRISK3 model, **Model 1** – Updated model with QRISK3 predictors only, **Model 2a** – Updated model with QRISK3 linear predictor plus additional reproductive and pregnancy complications, **Model 2b** – Updated model with all predictors (QRISK3 predictors plus reproductive and pregnancy complications.

#### Fig. S7f: Pre-eclampsia

**QRISK3** – original QRISK3 model, **QRISK3-R** – Recalibrated QRISK3 model, **Model 1** – Updated model with QRISK3 predictors only, **Model 2a** – Updated model with QRISK3 linear predictor plus additional reproductive and pregnancy complications, **Model 2b** – Updated model with all predictors (QRISK3 predictors plus reproductive and pregnancy complications.

#### Fig. S7g: GDM

**QRISK3** – original QRISK3 model, **QRISK3-R** – Recalibrated QRISK3 model, **Model 1** – Updated model with QRISK3 predictors only, **Model 2a** – Updated model with QRISK3 linear predictor plus additional reproductive and pregnancy complications, **Model 2b** – Updated model with all predictors (QRISK3 predictors plus reproductive and pregnancy complications.

#### Fig. S7h: Preterm birth

**QRISK3** – original QRISK3 model, **QRISK3-R** – Recalibrated QRISK3 model, **Model 1** – Updated model with QRISK3 predictors only, **Model 2a** – Updated model with QRISK3 linear predictor plus additional reproductive and pregnancy complications, **Model 2b** – Updated model with all predictors (QRISK3 predictors plus reproductive and pregnancy complications.

#### Fig. S7i: Stillbirth

**QRISK3** – original QRISK3 model, **QRISK3-R** – Recalibrated QRISK3 model, **Model 1** – Updated model with QRISK3 predictors only, **Model 2a** – Updated model with QRISK3 linear predictor plus additional reproductive and pregnancy complications, **Model 2b** – Updated model with all predictors (QRISK3 predictors plus reproductive and pregnancy complications.

#### Fig. S7j: Placental abruption

**QRISK3** – original QRISK3 model, **QRISK3-R** – Recalibrated QRISK3 model, **Model 1** – Updated model with QRISK3 predictors only, **Model 2a** – Updated model with QRISK3 linear predictor plus additional reproductive and pregnancy complications, **Model 2b** – Updated model with all predictors (QRISK3 predictors plus reproductive and pregnancy complications.

#### Fig. S7k: SGA

**QRISK3** – original QRISK3 model, **QRISK3-R** – Recalibrated QRISK3 model, **Model 1** – Updated model with QRISK3 predictors only, **Model 2a** – Updated model with QRISK3 linear predictor plus additional reproductive and pregnancy complications, **Model 2b** – Updated model with all predictors (QRISK3 predictors plus reproductive and pregnancy complications.

#### Fig. S7l: Postnatal depression

**QRISK3** – original QRISK3 model, **QRISK3-R** – Recalibrated QRISK3 model, **Model 1** – Updated model with QRISK3 predictors only, **Model 2a** – Updated model with QRISK3 linear predictor plus additional reproductive and pregnancy complications, **Model 2b** – Updated model with all predictors (QRISK3 predictors plus reproductive and pregnancy complications.

## Calibration plots overall and by subgroup in those aged 25 years and above

### Fig. S8: Overall calibration plot

**QRISK3** – original QRISK3 model, **QRISK3-R** – Recalibrated QRISK3 model, **Model 1** – Updated model with QRISK3 predictors only, **Model 2a** – Updated model with QRISK3 linear predictor plus additional reproductive and pregnancy complications, **Model 2b** – Updated model with all predictors (QRISK3 predictors plus reproductive and pregnancy complications.

### Fig. S9: Age groups

Fig. S9a: Age 25 - 34

**QRISK3** – original QRISK3 model, **QRISK3-R** – Recalibrated QRISK3 model, **Model 1** – Updated model with QRISK3 predictors only, **Model 2a** – Updated model with QRISK3 linear predictor plus additional reproductive and pregnancy complications, **Model 2b** – Updated model with all predictors (QRISK3 predictors plus reproductive and pregnancy complications.

Fig. S9b: Age 35 – 49

**QRISK3** – original QRISK3 model, **QRISK3-R** – Recalibrated QRISK3 model, **Model 1** – Updated model with QRISK3 predictors only, **Model 2a** – Updated model with QRISK3 linear predictor plus additional reproductive and pregnancy complications, **Model 2b** – Updated model with all predictors (QRISK3 predictors plus reproductive and pregnancy complications.

### Fig. S10: Ethnicity

Fig. S10a: White

**QRISK3** – original QRISK3 model, **QRISK3-R** – Recalibrated QRISK3 model, **Model 1** – Updated model with QRISK3 predictors only, **Model 2a** – Updated model with QRISK3 linear predictor plus additional reproductive and pregnancy complications, **Model 2b** – Updated model with all predictors (QRISK3 predictors plus reproductive and pregnancy complications.

**Fig. S10b: Black ethnicity**

**QRISK3** – original QRISK3 model, **QRISK3-R** – Recalibrated QRISK3 model, **Model 1** – Updated model with QRISK3 predictors only, **Model 2a** – Updated model with QRISK3 linear predictor plus additional reproductive and pregnancy complications, **Model 2b** – Updated model with all predictors (QRISK3 predictors plus reproductive and pregnancy complications.

Fig. S10c: Asian ethnicity

**QRISK3** – original QRISK3 model, **QRISK3-R** – Recalibrated QRISK3 model, **Model 1** – Updated model with QRISK3 predictors only, **Model 2a** – Updated model with QRISK3 linear predictor plus additional reproductive and pregnancy complications, **Model 2b** – Updated model with all predictors (QRISK3 predictors plus reproductive and pregnancy complications.

Fig. S10d: Mixed ethnicity

**QRISK3** – original QRISK3 model, **QRISK3-R** – Recalibrated QRISK3 model, **Model 1** – Updated model with QRISK3 predictors only, **Model 2a** – Updated model with QRISK3 linear predictor plus additional reproductive and pregnancy complications, **Model 2b** – Updated model with all predictors (QRISK3 predictors plus reproductive and pregnancy complications.

**Fig. S10e: Other ethnicity**

**QRISK3** – original QRISK3 model, **QRISK3-R** – Recalibrated QRISK3 model, **Model 1** – Updated model with QRISK3 predictors only, **Model 2a** – Updated model with QRISK3 linear predictor plus additional reproductive and pregnancy complications, **Model 2b** – Updated model with all predictors (QRISK3 predictors plus reproductive and pregnancy complications.

Fig. S10f: Unknown ethnicity

**QRISK3** – original QRISK3 model, **QRISK3-R** – Recalibrated QRISK3 model, **Model 1** – Updated model with QRISK3 predictors only, **Model 2a** – Updated model with QRISK3 linear predictor plus additional reproductive and pregnancy complications, **Model 2b** – Updated model with all predictors (QRISK3 predictors plus reproductive and pregnancy complications.

### Fig. S11: Reproductive and pregnancy complications

#### Fig. S11a: PCOS

**QRISK3** – original QRISK3 model, **QRISK3-R** – Recalibrated QRISK3 model, **Model 1** – Updated model with QRISK3 predictors only, **Model 2a** – Updated model with QRISK3 linear predictor plus additional reproductive and pregnancy complications, **Model 2b** – Updated model with all predictors (QRISK3 predictors plus reproductive and pregnancy complications.

#### Fig. S11b: Irregular menses

**QRISK3** – original QRISK3 model, **QRISK3-R** – Recalibrated QRISK3 model, **Model 1** – Updated model with QRISK3 predictors only, **Model 2a** – Updated model with QRISK3 linear predictor plus additional reproductive and pregnancy complications, **Model 2b** – Updated model with all predictors (QRISK3 predictors plus reproductive and pregnancy complications.

#### Fig. S11c: Endometriosis

**QRISK3** – original QRISK3 model, **QRISK3-R** – Recalibrated QRISK3 model, **Model 1** – Updated model with QRISK3 predictors only, **Model 2a** – Updated model with QRISK3 linear predictor plus additional reproductive and pregnancy complications, **Model 2b** – Updated model with all predictors (QRISK3 predictors plus reproductive and pregnancy complications.

#### Fig. S11d: Miscarriage

**QRISK3** – original QRISK3 model, **QRISK3-R** – Recalibrated QRISK3 model, **Model 1** – Updated model with QRISK3 predictors only, **Model 2a** – Updated model with QRISK3 linear predictor plus additional reproductive and pregnancy complications, **Model 2b** – Updated model with all predictors (QRISK3 predictors plus reproductive and pregnancy complications.

#### Fig. S11e: Gestational hypertension

**QRISK3** – original QRISK3 model, **QRISK3-R** – Recalibrated QRISK3 model, **Model 1** – Updated model with QRISK3 predictors only, **Model 2a** – Updated model with QRISK3 linear predictor plus additional reproductive and pregnancy complications, **Model 2b** – Updated model with all predictors (QRISK3 predictors plus reproductive and pregnancy complications.

#### Fig. S11f: Pre-eclampsia

**QRISK3** – original QRISK3 model, **QRISK3-R** – Recalibrated QRISK3 model, **Model 1** – Updated model with QRISK3 predictors only, **Model 2a** – Updated model with QRISK3 linear predictor plus additional reproductive and pregnancy complications, **Model 2b** – Updated model with all predictors (QRISK3 predictors plus reproductive and pregnancy complications.

#### Fig. S11g: GDM

**QRISK3** – original QRISK3 model, **QRISK3-R** – Recalibrated QRISK3 model, **Model 1** – Updated model with QRISK3 predictors only, **Model 2a** – Updated model with QRISK3 linear predictor plus additional reproductive and pregnancy complications, **Model 2b** – Updated model with all predictors (QRISK3 predictors plus reproductive and pregnancy complications.

#### Fig. S11h: Preterm birth

**QRISK3** – original QRISK3 model, **QRISK3-R** – Recalibrated QRISK3 model, **Model 1** – Updated model with QRISK3 predictors only, **Model 2a** – Updated model with QRISK3 linear predictor plus additional reproductive and pregnancy complications, **Model 2b** – Updated model with all predictors (QRISK3 predictors plus reproductive and pregnancy complications.

#### Fig. S11i: Stillbirth

**QRISK3** – original QRISK3 model, **QRISK3-R** – Recalibrated QRISK3 model, **Model 1** – Updated model with QRISK3 predictors only, **Model 2a** – Updated model with QRISK3 linear predictor plus additional reproductive and pregnancy complications, **Model 2b** – Updated model with all predictors (QRISK3 predictors plus reproductive and pregnancy complications.

#### Fig. S11j: Placental abruption

**QRISK3** – original QRISK3 model, **QRISK3-R** – Recalibrated QRISK3 model, **Model 1** – Updated model with QRISK3 predictors only, **Model 2a** – Updated model with QRISK3 linear predictor plus additional reproductive and pregnancy complications, **Model 2b** – Updated model with all predictors (QRISK3 predictors plus reproductive and pregnancy complications.

#### Fig. S11k: SGA

**QRISK3** – original QRISK3 model, **QRISK3-R** – Recalibrated QRISK3 model, **Model 1** – Updated model with QRISK3 predictors only, **Model 2a** – Updated model with QRISK3 linear predictor plus additional reproductive and pregnancy complications, **Model 2b** – Updated model with all predictors (QRISK3 predictors plus reproductive and pregnancy complications.

#### Fig. S11l: Postnatal depression

**QRISK3** – original QRISK3 model, **QRISK3-R** – Recalibrated QRISK3 model, **Model 1** – Updated model with QRISK3 predictors only, **Model 2a** – Updated model with QRISK3 linear predictor plus additional reproductive and pregnancy complications, **Model 2b** – Updated model with all predictors (QRISK3 predictors plus reproductive and pregnancy complications.

## Calibration in sensitivity analysis based on multiple imputation

### Fig. S12: Calibration plot in sensitivity analysis based on multiple imputation

## Clinical utility in complete case analysis

### Fig. S13: Clinical utility in complete case analysis

## Clinical utility in those aged 25 years and above

### Fig. S14: Clinical utility

## Clinical utility in sensitivity analysis based on multiple imputation

### Fig. S15: Clinical utility
